# Comparative Genomics Identifies a Novel Conserved Protein, HpaT, in Proteobacterial Type III Secretion Systems that Do Not Possess the Putative Translocon Protein HrpF

**DOI:** 10.3389/fmicb.2017.01177

**Published:** 2017-06-26

**Authors:** Céline Pesce, Jonathan M. Jacobs, Edwige Berthelot, Marion Perret, Taca Vancheva, Claude Bragard, Ralf Koebnik

**Affiliations:** ^1^UMR 186 IRD-Cirad-Université Montpellier IPMEMontpellier, France; ^2^Applied Microbiology Phytopathology, Earth and Life Institute, Université catholique de LouvainLouvain-la-Neuve, Belgium

**Keywords:** *Xanthomonas translucens*, *Xanthomonas hyacinthi*, *Xanthomonas theicola*, *Collimonas fungivorans*, hrp genes, translocon, XopA protein, XopM

## Abstract

*Xanthomonas translucens* is the causal agent of bacterial leaf streak, the most common bacterial disease of wheat and barley. To cause disease, most xanthomonads depend on a highly conserved type III secretion system, which translocates type III effectors into host plant cells. Mutagenesis of the conserved type III secretion gene *hrcT* confirmed that the *X. translucens* type III secretion system is required to cause disease on the host plant barley and to trigger a non-host hypersensitive response (HR) in pepper leaves. Type III effectors are delivered to the host cell by a surface appendage, the Hrp pilus, and a translocon protein complex that inserts into the plant cell plasma membrane. Homologs of the *Xanthomonas* HrpF protein, including PopF from *Ralstonia solanacearum* and NolX from rhizobia, are thought to act as a translocon protein. Comparative genomics revealed that *X. translucens* strains harbor a noncanonical *hrp* gene cluster, which rather shares features with type III secretion systems from *Ralstonia solanacearum*, *Paraburkholderia andropogonis*, *Collimonas fungivorans*, and *Uliginosibacterium gangwonense* than other *Xanthomonas* spp. Surprisingly, none of these bacteria, except *R. solanacearum*, encode a homolog of the HrpF translocon. Here, we aimed at identifying a candidate translocon from *X. translucens*. Notably, genomes from strains that lacked *hrpF*/*popF*/*nolX* instead encode another gene, called *hpaT*, adjacent to and co-regulated with the type III secretion system gene cluster. An insertional mutant in the *X. translucens hpaT* gene, which is the first gene of a two-gene operon, *hpaT*-*hpaH*, was non-pathogenic on barley and did not cause the HR or programmed cell death in non-host pepper similar to the *hrcT* mutant. The *hpaT* mutant phenotypes were partially complemented by either *hpaT* or the downstream gene, *hpaH*, which has been described as a facilitator of translocation in *Xanthomonas oryzae*. Interestingly, the *hpaT* mutant was also complemented by the *hrpF* gene from *Xanthomonas euvesicatoria*. These findings reveal that both HpaT and HpaH contribute to the injection of type III effectors into plant cells.

## Introduction

Many Gram-negative plant-pathogenic bacteria including most *Xanthomonas* species rely on a highly conserved Type III protein secretion system (T3SS) for pathogenicity on host plants. The T3SS delivers effector proteins (T3Es) directly into host cells. T3Es are thought to be exported through a hollow Type III pilus independent of a classical cleavage-dependent signal peptide used by most other bacterial secretion systems (Büttner and Bonas, 2010). T3Es are known to suppress host defenses and alter plant physiology to enhance infection (Büttner, 2016).

Genes encoding the T3SS are typically clustered in a ∼20 kb genomic island. In plant pathogens, the T3SS genes have been collectively called the *hrp* [hypersensitive response (HR) and pathogenicity] gene cluster since mutants in these genes were found to be impaired in their ability to cause the HR or programmed cell death on non-host plants and lost pathogenicity on host plants (Büttner and He, 2009). The first *hrp* gene clusters from *Xanthomonas* have been described in 1991 for *Xanthomonas euvesicatoria* (a.k.a. *Xanthomonas campestris* pv. *vesicatoria*) and for *Xanthomonas campestris* pv. *campestris* (Arlat et al., 1991; Bonas et al., 1991). *hrp* genes have been further subcategorized according to their broad conservation and subtle phenotypes of some of the corresponding mutants. Eleven *hrp* genes that are conserved among plant and animal pathogens and which are critical for causing disease have been renamed into *hrc* (HR conserved) genes (Bogdanove et al., 1996). And those genes that contribute to but are not essential for the plant–bacteria interaction are called *hpa* (*hrp* associated) genes. The core *hrp* cluster consists of six *hrp* operons, *hrpA* to *hrpF*, encompassing a total of eleven *hrc*, seven *hrp*, and three *hpa* genes with identical genetic organization in other *Xanthomonas* strains (Weber et al., 2007).

Two different types of *hrp* gene clusters have been described for plant pathogens, based on their gene repertoires, operon structures and mode of regulation (Büttner and Bonas, 2002a). Group I comprises *Pseudomonas syringae* and plant-pathogenic enterobacteria while group II includes bacteria in the genera *Acidovorax* and *Xanthomonas* and the *Ralstonia solanacearum* species complex. Group-II *hrp* genes and their co-regulated T3E genes are under control of two key regulatory genes, *hrpG* and *hrpX* (*hrpB* in *R. solanacearum* and *Acidovorax*) (Büttner and Bonas, 2010). HrpG shares characteristics with two-component response regulators of the OmpR family and induces the expression of *hrpX*. HrpX is an AraC-type transcriptional activator that binds to the plant-inducible promoter (PIP) box (TTCGB-N_15_-TTCGB), which is present in front of most of the *hrp* operons and many T3E genes (Koebnik et al., 2006).

Expression of the *hrp* gene cluster results in the formation of the contiguous molecular syringe that spans the bacterial cell envelope (Büttner and He, 2009; Kay and Bonas, 2009). It is predicted to consist of a multi-ring structure that is embedded in both inner and outer membrane and linked to the ATPase complex in the cytosol (Lorenz et al., 2012). In contrast to animal pathogens with their needle complex, plant pathogens evolved a pilus-like structure (i.e., encoded by *Xanthomonas hrpE* gene) for protein delivery into the plant’s cells (Weber et al., 2005). Type III-secreted proteins were located near the Hrp pilus during their secretion, suggesting that Hrp pili serve as conduits for the translocation of T3Es (Romantschuk et al., 2001).

Entry into the host cells is a final step in protein delivery; for this purpose, animal pathogens have evolved a multi-protein pore-forming translocon complex consisting of YopB and YopD, or homologs thereof (Chatterjee et al., 2013). However, no homologs of YopB or YopD were found in plant pathogens. Based on genetics and biophysical experiments, HrpF was identified to fulfill the role of a translocon protein in *Xanthomonas* (Rossier et al., 2000; Büttner and Bonas, 2002b); similarly, its homolog PopF is required for protein delivery from *R. solanacearum* (Meyer et al., 2006).

To date, the largest and the most conserved clade of xanthomonads (clade-2) includes the well-studied model species *X. euvesicatoria*, *X. campestris*, and *X. oryzae* (Parkinson et al., 2007). All functional studies of the *Xanthomonas hrp* cluster have been performed in this group. By far most strains of this *Xanthomonas* clade contain a canonical *hrp* cluster with the same genetic organization as in the model strains 85–10 and 8004 (Arlat et al., 1991; Bonas et al., 1991). Only recently researchers demonstrated that some clade-2 strains isolated from barley or cannabis are pathogenic despite the absence of an Hrp T3SS (Ignatov et al., 2015; Jacobs et al., 2015).

Much less work has been performed on members of the clade-1, which comprises the five highly diverse species *Xanthomonas albilineans*, *Xanthomonas sacchari, Xanthomonas theicola*, *Xanthomonas hyacinthi*, and *Xanthomonas translucens*. For two of them, *X. albilineans* and *X. sacchari*, genomic analyses demonstrated that they do not contain an Hrp T3SS (Pieretti et al., 2009; Studholme et al., 2011). In contrast, the 48 available genome sequences from seven different pathovars of *X. translucens* (Wichmann et al., 2013; Gardiner et al., 2014; Pesce et al., 2015a,b; Hersemann et al., 2016, 2017; Jaenicke et al., 2016; Peng et al., 2016) and draft genome sequence of *X. hyacinthi* (Naushad et al., 2015) revealed that all of them contain an Hrp T3SS the genetic organization of which is at variance to those from the clade-2.

*X. translucens* forms a diverse group of bacteria, most of which are pathogenic to monocotyledonous plants, such as various grasses and small-grain cereals. Strains have been assigned to ten pathovar subgroups based on symptoms on different host plants. Mutants in the *hrp* structural genes *hrcR* and *hrpE* and in the major regulator gene *hrpG* of the forage grass pathogen *X. translucens* pv. *graminis* were impaired in their ability to cause symptoms when compared with the wild-type strain *Xtg*29 (Wichmann et al., 2013). Notably, unlike other clade-2 *Xanthomonas* spp., the *X. translucens* pv. *graminis hrpG* mutant, the only mutant that was complemented in this study, still caused clearly visible symptoms, and complementation was only partially achieved. Moreover, bacterial colonization of the *hrp* mutants in the plant tissue was only slightly impaired, and all mutants were still quantified in comparable numbers similar to wild-type bacteria; whereas clade-2 *Xanthomonas hrp* mutants are typically severely reduced in colonization (Wichmann et al., 2013). Hence, these observations were in sharp contrast to those with clade-2 xanthomonads, thus casting doubt on the importance of the Hrp T3SS for the pathogenicity of *X. translucens*.

In this study, we therefore compared the core *hrp* gene clusters and their flanking sequences of several *X. translucens* strains with those from other bacterial pathogens. We analyzed them for novel genes that might be important for pathogenicity. Knockout mutagenesis of a conserved *hrc* gene demonstrated the importance of the Hrp T3SS for pathogenicity of barley-pathogenic *X. translucens* strains. Two other conserved genes, initially called *hgiA* and *hgiB* (for *hrpG*-induced gene), which are encoded next to the core *hrp* gene cluster, were analyzed for their contribution to pathogenicity of two pathovars of *X. translucens*. One of them, *hgiA*, was found to be important for pathogenicity on barley and to contribute to the non-host HR on pepper plants and was therefore re-named *hpaT* (for *hrp*-associated gene *T*). Given the absence of *hrpF* homologs in strains of *X. translucens*, we provide evidence that *hpaT* might encode an undescribed translocon component of the *X. translucens* T3SS.

## Materials and Methods

### Bacterial Strains and Growth Conditions

*Xanthomonas* strains used in this study are listed in **Table 1**. Strains were cultivated at 28°C in PSA medium (10 g peptone, 10 g sucrose, 1 g glutamic acid, 16 g agar, l^-1^ H_2_O). *Escherichia coli* DH10b bacteria (Durfee et al., 2008), which were used for molecular cloning, were cultivated at 37°C in lysogenic broth (LB).

**Table 1:** *Xanthomonas* strains and plasmids used in this study.

Bacterial strains and plasmids	Description	Reference
pVO155	pUC119-derived suicide vector (Km^R^)	Oke and Long, 1999
pVO155::*hrcT*	pVO155 containing a 419-bp internal *hrcT* fragment from CFBP 2054	This study
pVO155::*hpaT*	pVO155 containing a 410-bp internal *hpaT* fragment from CFBP 2054	This study
pVO155::*hgiB*	pVO155 containing a 430-bp internal *hgiB* fragment from CFBP 2054	This study
pBBR1MCS-5	Broad-host-range vector (Gm^R^)	Kovach et al., 1995
pHrcT	pBBR1MCS-5 containing *hrcT* from CFBP 2541	This study
pHpaT	pBBR1MCS-5 containing *hpaT* from CFBP 2541	This study
pHpaH	pBBR1MCS-5 containing *hpaH* from CFBP 2541	This study
pLHrpF	pLAFR3 containing the *hrpF* gene from *X. euvesicatoria* strain 85-10	Büttner et al., 2002
pBBR1MCS-5::*hrpG*^∗^	pBBR1MCS-5 containing the *hrpG^∗^* allele from *X. euvesicatoria* strain 85-10, which renders *hrp* gene expression constitutive in rich medium	Jacobs et al., 2015
pBBR1MCS-5::*hrpX*	pBBR1MCS-5 containing the *hrpX* gene from *X. euvesicatoria* strain 85-10	Koebnik et al., 2006

CFBP 2054^R^	*X. translucens* pv. *translucens* (Rf^R^)	This study
CFBP 2541^R^	*X. translucens* pv. *cerealis* (Rf^R^)	Pesce et al., 2015b
UPB787^R^	*X. translucens* pv. *translucens* (Rf^R^)	This study
UPB820^R^	*X. translucens* pv. *hordei* (Rf^R^)	This study
UPB820^R^ (pBBR1MCS-5)	UPB820^R^ containing the empty vector pBBR1MCS-5	This study
UPB820^R^ (pBBR1MCS-5::*hrpG*^∗^)	UPB820^R^ containing pBBR1MCS-5::*hrpG*^∗^	This study
UPB820^R^ (pBBR1MCS-5::*hrpX*)	UPB820^R^ containing pBBR1MCS-5::*hrpX*	This study
UPB787^R^ *hrcT^-^*	UPB787^R^ with pVO155 inserted into *hrcT*	This study
UPB820^R^ *hrcT^-^*	UPB820^R^ with pVO155 inserted into *hrcT*	This study
UPB787^R^ *hpaT^-^*	UPB787^R^ with pVO155 inserted into *hpaT*	This study
UPB820^R^ *hpaT^-^*	UPB820^R^ with pVO155 inserted into *hpaT*	This study
UPB787^R^ *hgiB^-^*	UPB787^R^ with pVO155 inserted into *hgiB*	This study
UPB820^R^ *hgiB^-^*	UPB820^R^ with pVO155 inserted into *hgiB*	This study
UPB787^R^ *hrcT^-^* (ev)	UPB787^R^ *hrcT^-^* with pBBR1MCS-5 (empty vector)	This study
UPB787^R^ *hrcT^-^* (pHrcT)	UPB787^R^ *hrcT^-^* complemented with pHrcT	This study
UPB787^R^ *hpaT^-^* (pHpaT)	UPB787^R^ *hpaT^-^* complemented with pHpaT	This study
UPB787^R^ *hpaT^-^* (pHpaH)	UPB787^R^ *hpaT^-^* complemented with pHpaH	This study
UPB787^R^ *hpaT^-^* (pLHrpF)	UPB787^R^ *hpaT^-^* complemented with pLHrpF	This study
UPB820^R^ *hpaT^-^* (pHpaT)	UPB820^R^ *hpaT^-^* complemented with pHpaT	This study
UPB820^R^ *hpaT^-^* (pHpaH)	UPB820^R^ *hpaT^-^* complemented with pHpaH	This study
85-10	*X. euvesicatoria* (Rf^R^)	Thieme et al., 2005
85-10 *hrpF*^-^	85-10 with pOK1 inserted into *hrpF*	Büttner et al., 2002
85-10 *hrpF*^-^ (pHpaT)	85-10 *hrpF ^-^* complemented with pHpaT	This study

*Resistances to the antibiotics kanamycin, gentamycin and rifampicin are annotated as Km^*R*^, Gm^*R*^, and Rf^*R*^, respectively*.

Rifampicin-resistant *Xanthomonas* mutants were selected upon plating on rifampicin-containing PSA medium at high cell density and one clone was chosen for further experiments. Plasmids were introduced into *E. coli* by thermo-transformation and into *X. translucens* by conjugation using pRK2013 as a helper plasmid in tri-parental mating (Figurski and Helinski, 1979). Antibiotics were added to the medium at the following final concentrations: rifampicin, 100 μg/ml; gentamicin, 20 μg/ml; kanamycin, 50 μg/ml.

### Plant Material and Plant Inoculations

All plants (barley and pepper) were grown in growth chambers with cycles of 16 hours of light per day at 22°C and 50% relative humidity.

Plants of the barley (*Hordeum vulgare* L.) cultivar Morex (six-rowed spring barley) were used for virulence assays and to follow the bacterial colonization *in planta*. For inoculation of barley leaves, three-week old plants were cut at about two centimeters below the leaf tip with sterile scissors that have been soaked in a bacterial suspension at optical density at 600 nm (OD_600_) of 0.5, corresponding to 3 × 10^8^ CFU/mL, and symptom development was followed over time (until 15 days post-inoculation). Immediately after inoculation, plants were transferred for 24 h into a chamber providing nearly saturated relative humidity. Disease symptoms were assessed using five replications per condition. Statistical significance of the results was evaluated using the Student’s *t*-test. Symptom development was assayed 10 days post-inoculation. Water was used as negative control for all inoculation experiments.

For bacterial quantification in barley leaves, leaves of 4-week old barley plants were infiltrated with a bacterial suspension of *X. translucens* at an OD_600_ of 0.2 using a needleless syringe. One square centimeter leaf segments were collected six hours, two days and five days after infiltration, and ground into a fine powder using the Qiagen TissueLyser system (30 rps for 30 s). Ground material was resuspended in 500 μl of 10 mM MgCl_2_, and 5-μl drops of a tenfold dilution series were spotted as triplicates onto selective PSA plates containing rifampicin. Three technical replicates were done for each of the three biological replicates.

To monitor the ability to trigger a non-host HR, pepper variety ECW-10R was used (Kousik and Ritchie, 1999). Bacterial suspensions at an OD_600_ of 0.4 were infiltrated into the leaves of eight-week old pepper plants using a needleless syringe and leaves were scored for an HR at two to eight days after inoculation.

### Molecular Cloning Techniques and Construction of Mutants

Plasmid DNA was isolated using the Wizard^®^ Plus SV Minipreps DNA Purification System (Promega, United States). Restriction enzymes were used according to the manufacturer’s recommendations (New England Biolabs, United States). Cloning reactions were performed using a commercial ligation kit (Thermo Fisher Scientific, United States). Polymerase chain reactions (PCR) were conducted in 20 μL volumes using GoTaq^®^ G2 Polymerase (Promega, United States). Cells of *E. coli* were transformed with plasmid DNA following a thermal shock and the resulting clones were validated by PCR and DNA sequencing. Oligonucleotide sequences are provided in Supplementary Table S1.

Chromosomal knockout mutants in *X. translucens* were obtained upon introduction of the suicide vector pVO155 (Oke and Long, 1999) that contained an internal fragment of the target gene. Consequently, single crossing-over events via homologous recombination at the target gene led to gene disruptions. Mutations were confirmed by PCR and DNA sequencing. For primer design, the draft genome sequence of *X. translucens* pv. *translucens* strain DSM 18974 (GenBank accession number CAPJ01000000) was used because of its phylogenetic proximity to other strains of the same pathovar and to strains of the pathovar *hordei*. To knockout the *hrcT* gene, a conserved and essential component of the T3SS, a 419-bp DNA fragment was PCR amplified from genomic DNA of strain CFBP 2054 (a sibling of strain DSM 18974) with oligonucleotide primers containing unique restriction sites for XbaI and BamHI at their 5′ ends, thus facilitating subsequent cloning into pVO155. Similarly, 410-bp and 430-bp DNA fragments were amplified from genomic DNA of strain CFBP 2054 and subsequently cloned as XbaI-BamHI fragments into pVO155 to create knockouts in *hpaT* and *hgiB*, respectively.

Mutants were complemented using the medium-copy plasmid pBBR1MCS-5 (Kovach et al., 1995) as a vector into which the corresponding DNA fragments were cloned upon PCR amplification from the sequenced *X. translucens* pv. *cerealis* pathotype strain CFBP 2541 (Pesce et al., 2015b). All plasmid constructs were checked by PCR, sequenced and introduced into *X. translucens* strains by conjugation. For complementation of *hrcT*, an 825-bp DNA fragment was amplified and cloned using the restriction enzymes SalI and EcoRI. To complement the *hpaT* mutation, which was expected to have a polar effect on the downstream *hpaH* gene, a 1347-bp *hpaT* fragment (start to stop codon) was cloned using HindIII and XbaI. Similarly, a 684-bp *hpaH* fragment (start to stop codon) was cloned into pBBR1MCS-5 using HindIII and XbaI.

### Expression Analyses

Relative abundance of transcripts was assessed by quantitative reverse transcription PCR (qRT-PCR). Bacteria were grown overnight in liquid NB (Sigma–Aldrich, United States) supplemented with gentamycin (20 μg l^-1^) and transferred to fresh 10 mL NB media with gentamycin for a final OD_600_ = 0.5. Bacteria were incubated for 3 h, shaking at 28°C. Transcriptional profiles and RNA was preserved with 5% phenol in ethanol as previously described (Jacobs et al., 2015). Bacterial RNA was extracted with Trizol (Invitrogen, United States), cleaned up with Zymogen RNA concentrator (Zymo Research, United States) and treated with Turbo DNase (Invitrogen, United States) following manufacturer’s protocols. RNA (1 μg per sample) was reverse transcribed with Superscript III (Invitrogen, United States) following the manufacturer’s recommendation. qPCR with SYBR MESA BLUE MasterMix (Eurogentec, Belgium) was performed following the manufacturer’s protocol on a Roche LightCycler 480 Real-Time PCR instrument (Roche Diagnostics Corporation, United States) with reaction parameters of 10-min polymerase activation at 95°C, then 40 cycles, with an individual cycle consisting of 15 s at 95°C and 1 min at 60°C.

### Bioinformatic Analyses

Database searches were performed using BLAST or PSI-BLAST (Altschul et al., 1990, 1997) For PSI-BLAST searches at NCBI^1^, hits with e-values smaller than 10^-10^ were used for iterative cycles. Multiple sequence alignments were generated using MUSCLE (Edgar, 2004) at the European Bioinformatics Institute^2^. Global sequence alignments were calculated at https://blast.ncbi.nlm.nih.gov/Blast.cgi (Needleman and Wunsch, 1970). The Artemis genome browser at http://www.sanger.ac.uk/resources/software/artemis/ was used to (re)annotate genomic regions of interest, such as the *hrp* clusters (Rutherford et al., 2000). The consensus sequence logo was generated at the website http://weblogo.berkeley.edu/logo.cgi (Crooks et al., 2004). Transmembrane alpha helices were predicted by the following algorithms: the well-known programs TMHMM v.2.0 at http://www.cbs.dtu.dk/services/TMHMM/ (Krogh et al., 2001) and Phobius at http://phobius.sbc.su.se/ (Käll et al., 2007), and the TOPCONS consensus prediction method at http://topcons.net (Tsirigos et al., 2015).

## Results

### Clade-1 Strains of *Xanthomonas* Harbor a Non-canonical *hrp* Cluster with Features Similar to T3SS Clusters from Betaproteobacteria

**FIGURE 1:**
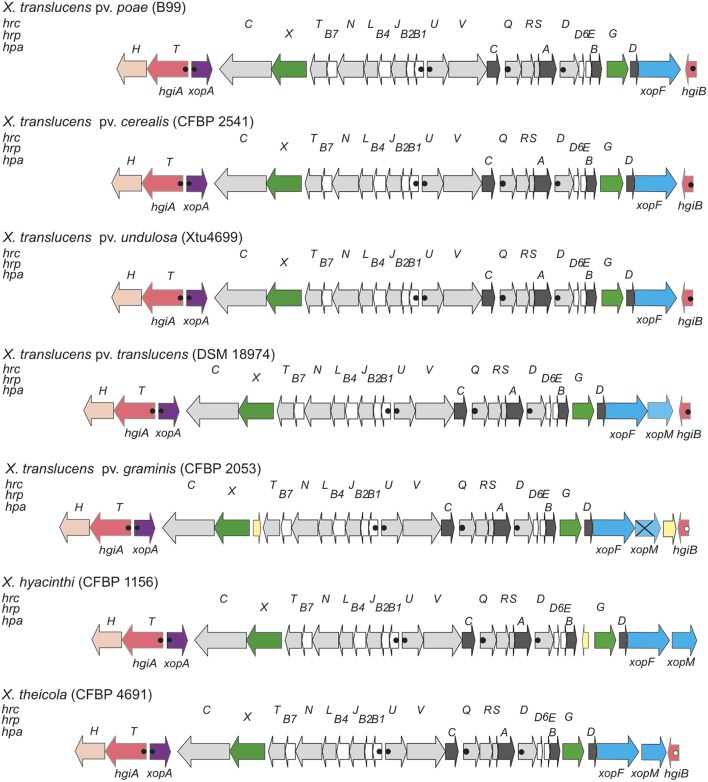
Genetic organization of the *hrp* gene cluster of clade-1 xanthomonads. Schematic overview of the *hrp* gene cluster and flanking regions from *Xanthomonas translucens* pathovars *poae* (strain B99), *cerealis* (strain CFBP 2541), *undulosa* (strain Xtu4699), *translucens* (strain DSM 18974), and *graminis* (strain CFBP 2053), and from two additional clade-1 xanthomonads, *X. hyacinthi* (strain CFBP 1156), and *X. theicola* (strain CFBP 4691). Conserved *hrc*, *hrp* and *hpa* genes are represented by light gray arrows, white arrows and dark gray arrows, respectively. Genes for type III effectors, key regulators, *hpaT* and *hgiB* genes, the *xopA* gene, the *hpaH* gene and mobile genetic elements are represented by blue arrows, green arrows, salmon arrows, lilac arrows, peach arrows and yellow arrows, respectively. Canonical plant-inducible promoters (PIP) are indicated by filled dots. Open dots correspond to noncanonical PIPs with minor deviations from the consensus sequence or in the spacer lengths between the conserved sequence motifs. Distances between operons are not drawn to scale. The crossed arrow for *X. translucens* pv. *graminis* CFBP 2053 corresponds to a frame-shifted variant of the *xopM* T3E gene.

To better understand the structure and molecular function of the *X. translucens* T3SS, we compared *hrp* clusters from five distinct pathovars (**Figure 1**). In *X. translucens*, the *hrpX* gene is always present downstream of *hrcT* and upstream of *hrcC*. *hrpX* and *hrcC* may form an operon because their coding sequences are only separated by 11 base pairs, which harbor a canonical Shine-Dalgarno sequence, AGGAGG, 4 bp in front of the ATG start codon of *hrcC*. In contrast, clade-2 xanthomonads have their *hrcC* gene as a single gene downstream of the last gene of the *hrpB* operon, *hrcT*. The second Hrp regulator of *X. translucens*, HrpG, is encoded at the other side of the *hrp* cluster, downstream of *hpaB*. This genetic organization of the *hrp* cluster is reminiscent of *R. solanacearum*, where *hrpX* is called *hrpB* (van Gijsegem et al., 1995; Brito et al., 1999). Most *hrp* operons were found to be preceded by a canonical PIP box and a properly spaced –10 promoter motif (**Figure 1**) (Koebnik et al., 2006).

A comparison of five genome sequences from different pathovars of *X. translucens* revealed that three genes, most likely belonging to two HrpX-controlled operons, are present downstream of *hrcC* (**Figure 1**). First, *xopA* (also known as *hpa1* in some clade-2 xanthomonads, such as *X. oryzae*) is found next to *hrcC*, but transcribed in opposite direction, and contains all elements of a plant-inducible promoter (PIP box and –10 motif), reminiscent of the genetic organization in clade-2 xanthomonads. Further downstream, another plant-inducible promoter is predicted to control the expression of an unknown gene, which we tentatively called *hgiA* for *hrpG*-induced gene A, followed by *hpaH* (syn. *hpa2*). Owing to the phenotype of the *hgiA* mutant (see below), the *hgiA* gene was later renamed *hpaT* (for *hrp*-associated gene *T*). In contrast to this genetic organization in clade-1 xanthomonads, *hpaH* and *xopA* are next to each other in clade-2 xanthomonads.

On the other edge of the core *hrp* cluster, *hrpG* is encoded downstream of *hpaB*, followed by the helper gene *hpaD* and its associated effector gene *xopF*. Further downstream, another unknown gene, which we call *hgiB*, is found that contains all elements of a plant-inducible promoter (except for strain CFBP 2053, which has one mismatch in the –10 motif). *X. translucens* pv. *translucens* strain DSM 18974 contains another T3E gene, *xopM*, between *xopF* and *hgiB*. Interestingly, this gene is also present in *X. translucens* pv. *graminis* strain CFBP 2053 where it, however, appears to be inactivated due to frameshift mutations.

In contrast to all other xanthomonads, *X. translucens* pv. *graminis* strains contain IS element remnants between *hrcT* and *hrpX*, which, however, are unlikely to affect the functionality of the T3SS. Another IS element was found to be inserted in *X. translucens* pv. *graminis* between the frameshifted *xopM* derivative and *hgiB*. We had access to the draft genome sequences of two more clade-1 species, *X. hyacinthi* strain CFBP 1156 and *X. theicola* strain CFBP 4691 (Jacques and co-workers, unpublished data). The genetic organization of the *hrp* clusters of these two clade-1 members appears to be identical to that of the *X. translucens hrp* clusters, except that some remnants of IS elements appear to separate *hpaB* from *hrpG* in strain CFBP 1156 (**Figure 1**).

**FIGURE 2:**
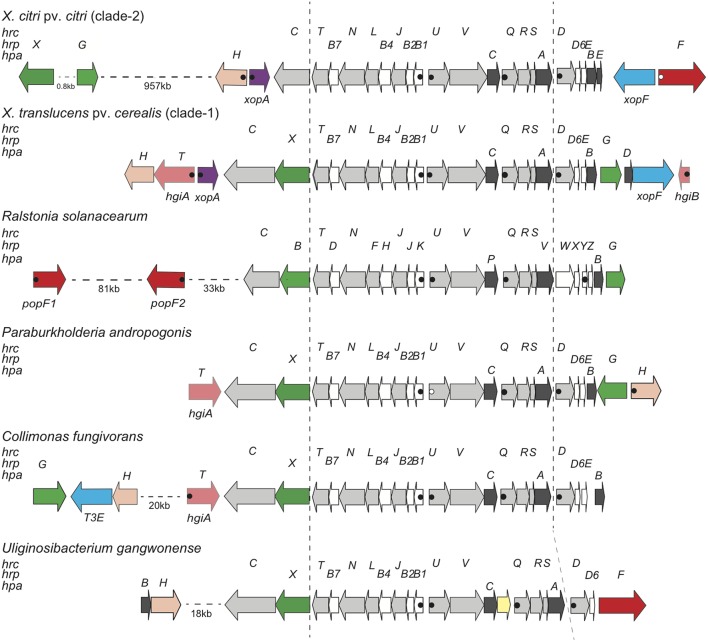
Genetic organization of the T3SS gene cluster from diverse Gram-negative bacteria. Schematic overview of the T3SS gene cluster and flanking regions from *X. citri* pv. *citri* (strain 306), *X. translucens* pv. *cerealis* (strain CFBP 2541), *R. solanacearum* (strain GMI1000), *P. andropogonis* (strain ICMP 2807), *C. fungivorans* (strain Ter331) and *U. gangwonense* (strain DSM 18521). Conserved *hrc*, *hrp* and *hpa* genes are represented by light gray arrows, white arrows and dark gray arrows, respectively. Genes for type III effectors, key regulators, putative translocons, *hpaT* and *hgiB* genes, the *xopA* gene, the *hpaH* gene and mobile genetic elements are represented by blue arrows, green arrows, red arrows, salmon arrows, lilac arrows, peach arrows and yellow arrows, respectively. Canonical PIPs are indicated by filled dots. Open dots correspond to noncanonical PIPs with minor deviations from the consensus sequence or in the spacer lengths between the conserved sequence motifs. Distances between operons are not drawn to scale.

**FIGURE 3:**
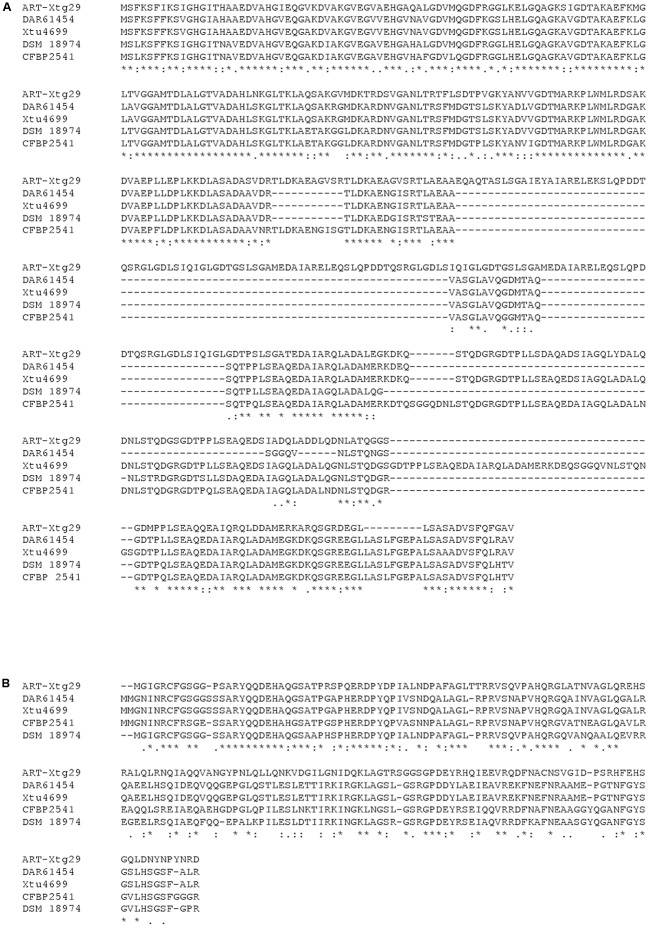
Sequence comparison of HpaT and HgiB proteins from *X. translucens*. Comparison of HpaT **(A)** and HgiB **(B)** amino acid sequences from five strains, extracted from their genome sequences (DAR61454, GenBank acc. no. AMXY00000000; DSM 18974, acc. no. CAPJ01000000; CFBP 2541, acc. no. JWHD00000000; ART-Xtg29, acc. no. ANGG00000000; Xtu4699, acc. no. CP008714).

Surprisingly, we did not find any homologs of the translocon protein HrpF (called PopF in *R. solanacearum*), which is encoded next to the *hrp* cluster in clade-2 xanthomonads and somewhere else in the genome of *R. solanacearum* (**Figure 2**). We also did not find any homologs of the few proteins from *Pseudomonas syringae* that have been shown to promote translocation of T3Es, namely Hrpk1, HrpW1, HrpZ1, and HopAK1 (Kvitko et al., 2007). Likewise, we did not find any homologs of the translocon components from human pathogens (LcrV-YopB-YopD from *Yersinia*, PcrV-PopB-PopD from *Pseudomonas*, SipB-SipC from *Salmonella*, IpaB-IpaC from *Shigella*) (Chatterjee et al., 2013). We therefore speculate that one of the unknown proteins that are encoded next to the *X. translucens* core *hrp* clusters, HpaT or HgiB, could play the role of a translocon. Multiple sequence alignments of both proteins revealed a surprisingly high level of divergence, a hallmark that these proteins share with other translocon proteins (**Figure 3**) (Büttner and Bonas, 2002a; Büttner et al., 2007). Interestingly, sequence comparison of HpaT proteins from *X. translucens* revealed the presence of repeated sequence motifs (Supplementary Figure S1). For instance, strain Xtu4699 has five copies of a 20-amino acid motif (D/Q-TP-L/P-LSEAQED-A/S-IA-R/G-QLADA), whereas a related sequence motif is present seven times in strain ART-Xtg29, four times in strain CFBP 2541, three times in strain DSM 18974 and only two times in strain DAR61454. Notably, no transmembrane alpha-helical segment was predicted for HpaT or HgiB by any of the tested algorithms (TMHMM v.2.0, Phobius, TOPCONS).

Further insight in the structure and molecular function of the T3SS was expected from a comparison with other, taxonomically distant bacteria. BLAST and PSI-BLAST database searches revealed similar T3SS exist in the genera *Paraburkholderia*, *Collimonas*, and *Uliginosibacterium* (**Figure 2**). Strains of these bacteria have the same order of genes from *hrcC* to *hrcD*. Downstream of *hrcD*, *Uliginosibacterium gangwonense* strain DSM 18521 has an *hrpD6* ortholog encoding an 81-amino acid protein, while the other two bacteria encode polypeptides of similar length, which are likely to be orthologs of *hrpD6*. Short open reading frames between *hrpD6* and *hpaB* may encode pilin-like proteins in *Collimonas fungivorans* strain Ter331 and *Paraburkholderia andropogonis* strain ICMP 2807. Hrp pilin proteins are intrinsically hard to identify due to their small size and because they are under diversifying selection (Guttman et al., 2006; Weber and Koebnik, 2006). BLAST searches did not detect an *hrpG* ortholog in the genome of *U. gangwonense* strain DSM 18521, whereas *P. andropogonis* contains *hrpG* next to *hpaB*, as in *X. translucens*, but in opposite direction. *C. fungivorans* has an *hrpG* ortholog ∼22 kb away from the *hrp* cluster in a region that appears to encode at least three T3Es, in addition to HpaH. Most interestingly, while *U. gangwonense* has an HrpF ortholog, but no homolog of HpaT, the other two bacteria contain HpaT orthologs but do not encode HrpF. This mutual exclusivity suggests that HrpF and HpaT may functionally substitute for each other.

### The *Xanthomonas translucens hrp* Cluster Is Required to Cause Disease on Barley and to Trigger a Non-host HR in Pepper Leaves

To test the functional role of the *hrp* cluster, knock-out mutants were constructed in *hrcT*, a conserved *hrp* gene, in strains of two *X. translucens* pathovars infecting barley. This gene was chosen since it is a relatively small gene and it is located at the end of an operon (**Figure 1**), thus facilitating complementation assays. Moreover, mutations in *hrcT* of clade-2 xanthomonads have been shown to result in a null phenotype, i.e., the mutants were unable to cause disease on host plants or to elicit an HR on non-host plants (Li et al., 2011b) or resistant host plants (Bonas et al., 1991; Fenselau and Bonas, 1995).

**FIGURE 4:**
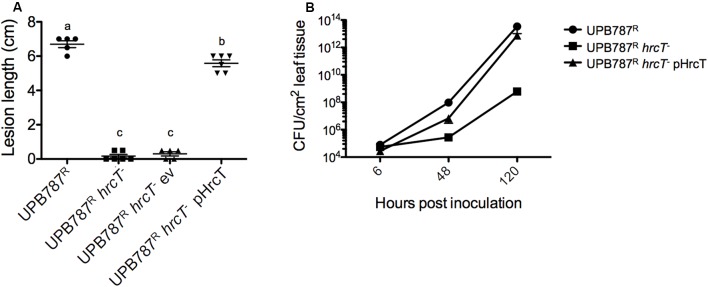
The T3SS is required for *X. translucens* pv. *translucens* symptom development and bacterial colonization of barley leaves. **(A)** The role of *hrcT* in *X. translucens* pv. *translucens* UPB787^R^ during plant pathogenesis was assessed by barley leaf clipping. Barley leaves were inoculated with the wild-type strain UPB787^R^ and the *hrcT* mutant (UPB787^R^ *hrcT*^-^), with the *hrcT* mutant containing an empty vector (UPB787^R^ *hrcT*^-^ ev) and with the *hrcT* mutant complemented with a plasmid-borne *hrcT* gene (UPB787R^R^ pHrcT). Lesion length was measured at 15 dpi. At least five plants were used per treatment. Lower case letter represents statistically significantly different treatments based on a Student’s *t*-test (*p* < 0.01). Treatments with the same letters are not statistically significantly different. Error bars represent the standard error of the mean. **(B)** Bacterial leaf colonization was quantified with dilution plating of leaves infiltrated with bacterial suspensions in water with the wild-type strain UPB787^R^, the *hrcT* mutant, and the *hrcT* mutant complement. Bacterial colonization was measured at six, 24 and 120 h post inoculation. Data are the mean of nine technical replicates per treatment. Error bars represent standard error of the mean.

To quantify the effect of the *hrcT* mutation on the progression of the disease, bacteria were inoculated by leaf clipping. Two weeks after inoculation, lesion lengths decreased from 6.7 cm for the wild-type UPB787^R^ to 0.2 cm for the *hrcT* mutant strains (**Figure 4A**). Similar results were obtained with UPB820^R^ and its *hrcT* mutant (Supplementary Figure S2). When complemented with a plasmid-borne *hrcT* gene from *X. translucens* pv. *translucens* strain CFBP 2054, both mutants fully regained the ability to cause symptoms (**Figure 4A** and Supplementary Figure S2). These data clearly demonstrate that the two *X. translucens* pathovars require *hrcT* for barley pathogenicity.

To assess the colonization of the plant tissue, barley leaves were infiltrated with the *hrcT* mutant of strain UPB787^R^ and its complemented derivative. Colonization of the leaf blade was monitored over a period of five days. Later time points were not taken into consideration because of tissue collapse. The UPB787^R^ *hrcT* mutant showed a significant reduction of bacterial growth at 2 and 5 days after inoculation when compared to the wild-type strain (**Figure 4B**). The complemented strain recovered the same population size as the wild type at five days after inoculation (**Figure 4B**), indicating that *hrcT* from T3SS of *X. translucens* pv. *translucens* is required for plant colonization.

**FIGURE 5:**
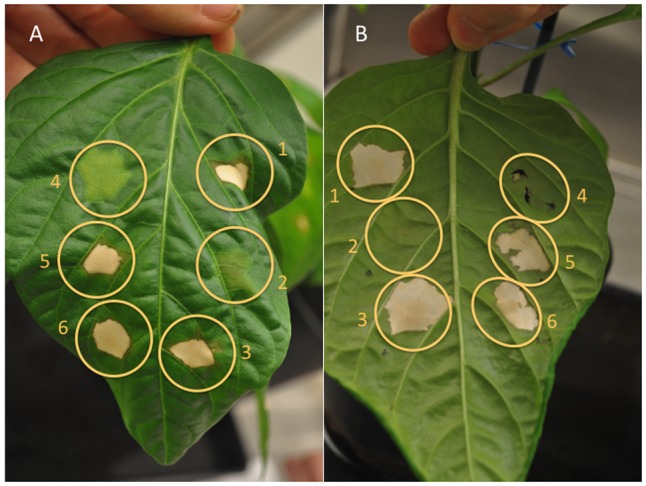
Three genes of the *X. translucens hrp* gene cluster, *hrcT*, *hpaT* and *hpaH*, contribute to full HR elicitation on non-host pepper plants. The role of *hrcT*, *hpaT* and *hpaH* in the *X. translucens* pathovars *translucens* (strain UPB787^R^, panel **(A)**, photo taken from the upper side of the leaf) and *hordei* (strain UPB820^R^, panel **(B)**, photo taken from the lower side of the leaf) was determined by pepper leaf infiltration. Leaves of the pepper cultivar ECW-10R were infiltrated with the wild-type strain (1), the *hrcT* mutant (2), the *hrcT* mutant complemented with *hrcT* gene (3), the *hpaT* mutant (4), the *hpaT* mutant complemented with the *hpaT* gene (5) or with the *hpaH* gene (6).

*X. translucens* pv. *translucens* strain UPB787^R^ was found to cause a non-host HR when inoculated into pepper leaves of the cultivar ECW-10R (**Figure 5A**). Since HR elicitation is typically associated with a functional Hrp T3SS, we inoculated the UPB787^R^ *hrcT* mutant and its complemented derivative onto pepper leaves. As expected, the mutant failed to trigger an HR while the complemented strain regained the ability to cause an HR similar to that caused by the wild type. As a marginal note, however, we would like to emphasize that the *hrcT* mutant appeared to be somewhat leaky in this assay since we occasionally observed a weak HR or little brown spots in the inoculated leaf area (**Figure 5A**). In conclusion, strains of cereal-pathogenic *X. translucens* require a functional Hrp system to cause a strong HR on non-host plants and to colonize the host plant barley. Similar results were obtained with the *X. translucens* pv. *hordei* strain UPB820^R^ (**Figure 5B**).

### Two Conserved Genes, *hpaT* and *hgiB*, are Co-expressed with the *hrp* Gene Cluster and *hpaT* Forms an Operon with *hpaH*

Comparison of five *X. translucens* strains had revealed the presence of two conserved, presumably HrpX-regulated genes, *hpaT* and *hgiB*, at the borders of their *hrp* clusters (**Figure 1**). Sequence alignments of the promoter regions (200 bp upstream of the presumed ATG start codon) of the five *X. translucens* strains shown in **Figure 1** with those of the two other clade-1 xanthomonads, *X. hyacinthi* and *X. theicola*, indicated the conservation of distinct *cis* regulatory elements corresponding to the PIP box and a properly spaced –10 motif (Supplementary Figure S3). The presence of such a canonical plant-inducible promoter sequence suggested that both genes are under direct transcriptional control of HrpX, the expression of which is controlled by HrpG (Koebnik et al., 2006).

Quantitative reverse transcription PCR (qRT-PCR) was used to determine the relative expression of the two putative HrpX-target genes in *X. translucens* strains UPB820^R^ pBBR1MCS-5::*hrpG*^∗^, UPB820^R^ pBBR1MCS-5::*hrpX* compared to UPB820^R^ pBBR1MCS-5 (empty vector) as a control. Two known *hrp* genes, *hrpB1* and *hrpC1* (syn. *hrcU*) were included for comparison. Both *hpaT* and *hgiB*, as well as the two *hrp* genes, were dramatically induced in UPB820^R^ ectopically expressing *hrpG*^∗^ or *hrpX*, compared to the empty vector control, as indicated by significantly lowered cycle treshold (Ct) values (Supplementary Table S2). We conclude that the promoters of *hpaT*, *hgiB*, *hrpB1*, and *hrpC1* are *bona fide* targets of HrpX, validating our bioinformatic analysis.

The genomic context of both genes indicated that *hgiB* is a single gene while *hpaT* could be the first gene of a two-gene operon with *hpaH* (**Figure 1**). Indeed, all *X. translucens* strains contained a conserved 5-bp sequence (CCCGT) between the TAG stop codon of *hpaT* and the putative ATG start codon of *hpaH* (Supplementary Figure S4). Similarly, the two coding sequences were separated by only four base pairs in *X. hyacinthi* and *X. theicola*. To test whether both genes are co-transcribed, we performed another qRT-PCR experiment where the forward primer matched to the 3’ end of *hpaT* and the reverse primer matched to the 5’ end of *hpaH*. Expession analyses in UPB820^R^ strains ectopically expressing *hrpG*^∗^ or *hrpX* confirmed that both genes are co-transcribed and that synthesis of both proteins is likely translationally coupled from a long transcript.

### The Conserved *hpaT* Gene Contributes to Symptom Development on Barley

Comparison of five *X. translucens* strains had revealed the presence of two conserved HrpX-regulated genes, *hpaT* and *hgiB*, at the borders of their *hrp* clusters. RNA-seq experiments had shown that these genes are also strongly induced in a *X. translucens* pv. *translucens* strain CFBP 2054^R^ that ectopically expresses a constitutively active form of the master regulator HrpG, called HrpG^∗^ (Wengelnik et al., 1999) from *X. euvesicatoria* 85–10 (unpublished data). In order to decipher a possible contribution of these two genes to disease, we created pVO155 insertion mutants in *hpaT* and *hgiB*. The genomic context of both genes indicated that *hgiB* is a single gene while *hpaT* is likely to be the first gene of a two-gene operon with *hpaH* (**Figure 1**). Consequently, the *hpaT* insertion mutant was expected to have a polar effect on the downstream *hpaH*.

**FIGURE 6:**
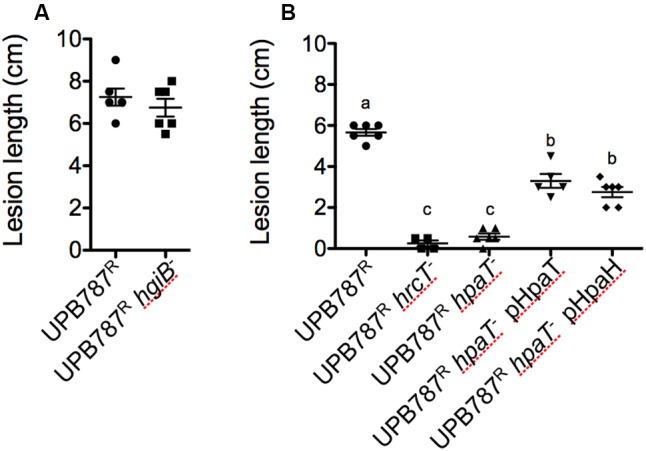
*Xanthomonas translucens* pv. *translucens* strain UPB787^R^ *hpaT* but not *hgiB* is required for pathogenicity on barley. The role of *hpaT* and *hgiB* in *X. translucens* pv. *translucens* UPB787^R^ was determined by barley leaf clipping. Barley leaves were inoculated with the wild-type strain UPB787^R^ and **(A)** the *hgiB* mutant (UPB787^R^ *hgiB^-^*) or **(B)** the *hrcT* mutant (UPB787^R^ *hrcT^-^*), the *hpaT* mutant (UPB787^R^ *hpaT^-^*), the *hpaT* mutant complemented with the *hpaT* gene (UPB787^R^ *hpaT^-^* pHpaT) or with the *hpaH* gene (UPB787^R^ *hpaT^-^* pHpaH). Lesion length was measured at 15 dpi. At least five plants were used per treatment. Lower case letter represents statistically significantly different treatments based on a Student’s *t*-test (*p* < 0.0001). Treatments with the same letters are not statistically significantly different. Error bars represent the standard error of the mean.

An insertion mutation in *hgiB* was constructed for *X. translucens* pv. *translucens* UPB787^R^. When the mutant was infiltrated into barley leaves, it behaved similar to the wild type (Supplementary Figure S5). Inoculation by leaf clipping did not demonstrate any differences between UPB787^R^ wild-type strains and its *hgiB* mutant (**Figure 6A**). A knock-out mutation in *hgiB* of the *hordei* pathovar, strain UPB820^R^, resulted again in symptoms similar to the wild type (Supplementary Figure S5). These experiments indicate that *hgiB* does not contribute to disease development on barley plants.

Next, the *hpaT* gene was knocked out in strain UPB787^R^. Leaf clip inoculations revealed that the UPB787^R^ *hpaT* mutant was strongly impaired in its ability to cause symptoms similar to that of the *hrcT* mutant (**Figure 6B**). Because *hpaT* and *hpaH* were in a putative operon, we individually cloned *hpaT* or *hpaH* into the broad-host range vector pBBR1MCS-5 and conjugated them into the mutant strains for complementation assays. Strains were inoculated by leaf clipping, and plants were scored 15 days after inoculation. Introducing *hpaT* into the UPB787^R^ *hpaT* insertion mutant caused a partial complementation, leading to 3.3 cm long lesions compared to the 5.7 cm long lesions caused by the wild type (**Figure 6B**). Surprisingly, we observed a slightly lower, but still significant level of complementation when only the *hpaH* gene was introduced into the UPB787^R^ *hpaT* mutant (**Figure 6B**). Similar results were obtained with the *hpaT* mutant and its complemented derivatives in the *X. translucens* pv. *hordei* strain UPB820^R^ (Supplementary Figure S6) as well as when we infiltrated bacteria into the leaf blade as a semi-quantitative assay (Supplementary Figure S7). These data suggest that both genes, *hpaT* and *hpaH*, contribute to virulence but none of them is absolutely required for causing symptoms.

### The HpaT Protein Contributes to the Non-host HR

The strong phenotype of the *hpaT* mutant suggested that the gene is either a key component for the delivery of T3Es into host cells, or it encodes itself a major virulence effector that contributes critically to disease. In the latter case we expected that a mutation in *hpaT* would not have a drastic effect on the ability to trigger a non-host HR, unless it is the HpaT protein itself that triggers an HR.

First, we tested the *X. translucens* pv. *translucens* UPB787^R^ *hpaT* mutant for HR elicitation on pepper plants of the cultivar ECW-10R (**Figure 5A**). While the wild-type strain triggered a strong HR, the mutant did not lead to any reaction, thus mirroring the phenotype of the *hrcT* mutant, which is unable to secrete effectors. When a plasmid-borne copy of *hpaT* was introduced into the UPB787^R^ *hpaT* mutant, an HR was observed, thus demonstrating the complementation of the mutant phenotype (**Figure 5A**). A similar HR was observed when we introduced *hpaH* on a plasmid into the UPB787^R^ *hpaT* insertion mutant. Similar results were obtained with the *X. translucens* pv. *hordei* strain UPB820^R^ and its mutants and complemented strains (**Figure 5B**).

### The HpaT Protein is Likely a Component of the *X. translucens* Translocon, Which Can Be Substituted by the *X. euvesicatoria* HrpF Protein

**FIGURE 7:**
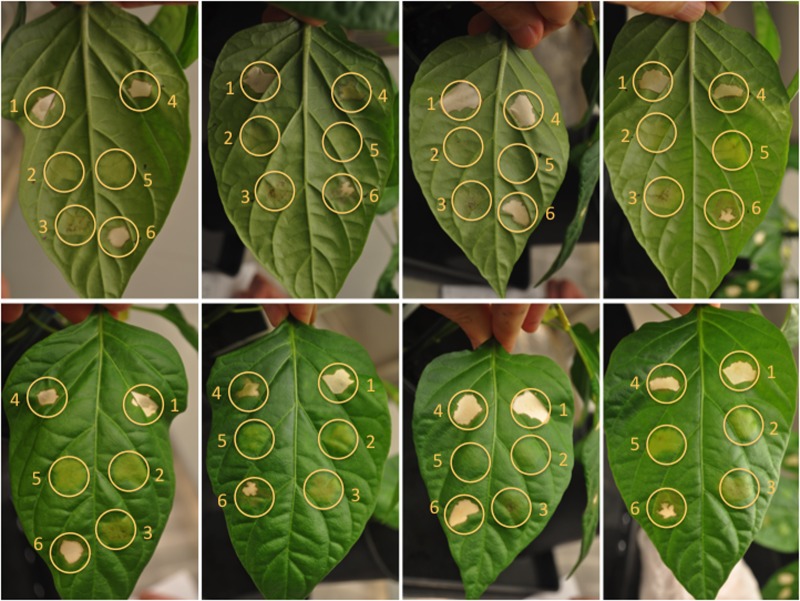
HpaT from *X. translucens* and HrpF from *X. euvesicatoria* are to some degree functionally interchangeable. The ability of trans-complementation between *hpaT* and *hrpF* was monitored by HR assays. Pepper leaves of the cultivar ECW-10R were inoculated with the wild-type strain 85–10 (1), the 85–10 *hrpF* mutant (2), the *hrpF* mutant complemented with *hpaT* (3), the wild-type strain UPB787^R^ (4), the UPB787^R^ *hpaT* mutant (5) or with the *hpaT* mutant complemented with *hrpF* (6). Four representative leaves were photographed one week after infiltration from the lower (top) and the upper (bottom) side of the leaf.

To decipher whether HpaT is indeed a new putative translocon component, we aimed at complementing the *hrpF* mutant of the *X. euvesicatoria* strain 85–10. Strain 85-10 contains the avirulence gene *avrBs1*, which is recognized by the pepper resistance gene *Bs1*. Inoculation of pepper leaves of the cultivar ECW-10R confirmed that *hrpF* is required to trigger the HR (**Figure 7**). When we introduced the *hpaT* gene on a plasmid, we observed in many cases some little brown spots in the area of inoculation (**Figure 7**). However, since the strength of this phenotype was weak and somehow variable, we are cautious to claim that *hpaT* can complement the *hrpF* mutant.

We next performed the reciprocal experiment, aiming at complementing the *hpaT* mutant with the *hrpF* gene from *X. euvesicatoria* strain 85–10. Surprisingly, the mutant phenotype was fully complemented when we inoculated ECW-10R pepper leaves with the mutated UPB787 strain that contained a plasmid-borne copy of *hrpF* (**Figure 7**). We take these results as evidence that HpaT and HrpF are functionally analogous proteins that lack detectable sequence homology.

## Discussion

The first *hrp* gene clusters from *Xanthomonas* were discovered in the clade-2 xanthomonads *X. euvesicatoria* and *X. campestris*, which rapidly became model organisms to study the pathogenicity of *Xanthomonas*. Other species, such as the rice pathogen *X. oryzae* and the citrus pathogen *X. citri* pv. *citri* were studied in detail as well due to their high economic interest and concerns about associated diseases, namely bacterial leaf blight and leaf streak of rice and citrus canker, respectively.

Collectively, data has accumulated for these species over the last 25 years. However, much less is known for species of the clade-1. *X. albilineans* GPE PC73 was the first clade-1 strain whose genome was sequenced (Pieretti et al., 2009), followed by strains of *X. sacchari* (Studholme et al., 2011). It came as a surprise that these clade-1 strains did not have an *hrp* gene cluster because it was known from previous work that at least pathovars of *X. translucens* contain *hrp* genes and T3Es of the TAL family (Alizadeh et al., 1997; Bragard et al., 1997). Indeed, when the first *X. translucens* genome was sequenced, a complete, but noncanonical *hrp* gene cluster was found (Wichmann et al., 2013). In contrast to clade-2 strains, however, a knockout in a conserved *hrp* gene of the grass pathogen *X. translucens* pv. *graminis* did not lead to the typically observed drastic loss of virulence (Wichmann et al., 2013).

In this study, we demonstrated that a mutation of the conserved *hrcT* gene of the barley pathogens *X. translucens* pv. *hordei* and *X. translucens* pv. *translucens* resulted in a loss of pathogenicity. Mutants were strongly attenuated in symptom formation and could hardly colonize the leaf blade upon leaf clip inoculation, a typical phenotype that is shared with clade-2 strains. Surprisingly *hrp* mutants in *hrcR* and *hrpE* and in the regulatory gene *hrpG* in grass pathogen *X. translucens* pv. *graminis* had little effect on virulence. This seems largely inconsistent with the wide importance of the T3SS structural components and regulators in *R. solanacearum*, clade-2 *Xanthomonas* spp. and *Pseudomonas syringae*. Perhaps T3Es are more import for the colonization of small-grain cereals than the colonization of grasses. It will therefore be interesting to broadly compare the same *hrp* mutants (e.g., *hrcT*) in other pathovars of *X. translucens*, such as the small-grain pathovars *undulosa* and the grass pathovars *arrhenateri*, *poae*, *phlei*, and *phleipratensis*. It will also be worthwhile to analyze the repertoires of T3Es of all these *X. translucens* pathovars.

A comparison of five *X. translucens hrp* gene clusters revealed that they all have the same genetic organization, which to some extend resembles that of the *hrp* cluster from *R. solanacearum*. In both species, the two key regulatory genes, *hrpG* and *hrpX*/*hrpB*, are encoded on the left and right side of the *hrp* cluster. In general, both flanking regions are largely conserved among the five *X. translucens* strains (**Figure 2**). One of the few differences was the presence of a large open reading frame (1587 bp) beyond *xopF* in the *X. translucens* pv. *translucens* strain DSM 18974, which is homolog to *xopM* from *X. euvesicatoria* (Schulze et al., 2012) and has homologs in other xanthomonads and in some betaproteobacteria, such as *Acidovorax*, *Collimonas*, *Ralstonia*, and *Rhizobacter*. A homologous sequence was also found at the same position in the two other clade-1 xanthomonads, *X. hyacinthi*, and *X. theicola*, and frameshifted derivatives were observed in strains of *X. translucens* pv. *graminis*. Inactivation of genes at the boundaries of *hrp* clusters has been observed for clade-2 xanthomonads and is often linked to T3E genes. For instance, *X. oryzae* pv. *oryzae* encodes the LRR effector XopAE (syn. HpaF) beyond *hrpF* while the corresponding gene in *X. euvesicatoria* 85–10 suffers from a frameshift mutation (Sugio et al., 2005). Likewise, the candidate chaperone gene *hpa3* in front of *hpa4* (syn. *xopF*) from *X. oryzae* pv. *oryzae* contains a frameshift in the *X. euvesicatoria* homolog (Sugio et al., 2005). The broadly conserved T3E XopM can be considered as a core effector, a finding that is substantiated by the observation that the *xopM* gene is typically encoded in close vicinity to the T3SS gene cluster, not only in *Xanthomonas* (e.g., next to *hpaH* in *X. euvesicatoria* strain 85–10 and *X. cannabis* pv. *phaseoli* strain Nyagatare), but also in the betaproteobacteria *Acidovorax* and *Collimonas*.

In addition to the atypical location of the *hrpG* and *hrpX* genes, we detected two other genes in the *hrp* regions that are conserved among strains of *X. translucens* and in *X. hyacinthi*. Both genes, *hpaT* and *hgiB*, are preceded by a canonical plant-inducible promoter sequence (Koebnik et al., 2006). Quantitative RT-PCR experiments with strain UPB820^R^ revealed that both genes are indeed strongly activated by HrpG (and HrpX). Therefore, these genes have tentatively been named *hgiA* and *hgiB*, *hgi* for HrpG-induced, following the nomenclature of Noël et al. (2001). Owing to the phenotype of the *hgiA* mutant, however, *hgiA* was renamed *hpaT*.

Both *hpaT* and *hgiB* show considerable sequence diversity among the analyzed *X. translucens* strains (**Figure 3**), a finding that let us speculate that the genes may be under diversifying selection and that the gene products may be exposed to the plant surveillance system. Yet, the small number of available sequences does not allow testing this hypothesis. Using BLAST and PSI-BLAST searches in NCBI GenBank, homologs of *hgiB* have only been detected in *X. translucens* while more distantly related genes of *hpaT* were also found in several betaproteobacteria, such as *Burkholderia*, *Collimonas*, *Massilia*, *Paraburkholderia*, and *Ralstonia*, where they are typically encoded in the vicinity of a T3SS gene cluster.

Insertional knockout mutants in *hgiB* did not show any defect in virulence when tested in two different strains. Apparently, this gene does not obviously contribute to the type III secretion and effector translocation. Further work will address the question if *hgiB* encodes another T3E. In contrast, when *hpaT* was knocked-out in two different strains by insertion of a plasmid which contains a partial *hpaT* sequence by single homologous recombination, the mutant bacteria was non-pathogenic on the barley variety Morex.

Moreover, we analyzed two strains of *X. translucens* that caused a non-host HR in the pepper cultivar ECW-10R. ECW-10R carries the *Bs1* resistance gene and produces an HR in response to the effector AvrBs1 from *X. campestris* pv. *vesicatoria* (Cook and Stall, 1963). However, neither UPB787^R^ nor UPB820^R^ has a homolog of *avrBs1* (GenBank acc. no. JTEM00000000, and data not shown). It is therefore likely that another, yet unidentified T3E is recognized by pepper.

HR elicitation by an *avr* gene depends on a functional T3SS, as shown by the phenotypes of the *hrcT* mutant. Full HR was also dependent on *hpaT* and *hpaH*. Altogether, the observed phenotypes with respect to *hpaT* and *hpaH* correspond to the typical phenotype of an *hrp* mutant, which is non-pathogenic on susceptible host plants and cannot cause a non-host HR. This strongly suggests that *hpaT* and *hpaH* contribute to the delivery of T3Es into plant cells.

Strains of *X. translucens* do not possess the translocon protein HrpF, which is ubiquitous in all clade-2 xanthomonads that have an Hrp system. Wichmann et al. (2013) speculated that cells of *X. translucens* pv. *graminis* may use breaches to gain direct access to the xylem cells in which they reside, and as these cells do not contain a cell membrane, the *hrpF* gene may be dispensable for effector translocation and, consequently, may have been lost during evolution. However, using GFP-labeled bacteria of *X. translucens* pv. *translucens* we could detect the bacteria in the vascular bundle upon leaf clipping, clearly demonstrating that the bacteria colonize the intercellular space of barley leaves (unpublished results). We therefore favor the second alternative that was put forward by Wichmann et al., namely that a distantly related *hrpF* gene (or better, an anolog of it) might be present, but cannot be recognized on the basis of sequence identity.

Comparison of T3SS gene clusters with a similar genetic organization and mode of regulation (via HrpX) from various bacteria revealed that all the strains that do not have a detectable *hrpF* gene do instead possess a homolog of *hpaT*. We took this mutual exclusivity as circumstantial evidence that *hpaT* might encode a functional analog of HrpF. No transmembrane alpha-helical segment was predicted for HpaT by any of the tested algorithms, a feature that is shared with other putative translocon proteins, HrpF from *X. euvesicatoria* and PopF1 and PopF2 from *R. solanacearum* (data not shown). Moreover, both protein families, the HrpF family and the HpaT family, share the presence of repeated sequence motifs as a common structural feature. HrpF from *X. campestris* pv. *campestris* has three imperfect repeats of ∼110 amino acids, whereas the prototype HrpF protein from *X. euvesicatoria* has only two repeats, and the corresponding homolog in *Sinorhizobium fredii*, NolX, has only one copy of the repeated sequences (Sugio et al., 2005). Another polymorphic region is found in the C-terminal region of HrpF proteins where 5 to 15 copies of an imperfect tetrapeptide repeat are present in different species of *Xanthomonas* (unpublished data). Similarly, HpaT proteins from *X. translucens* contain several repeated sequence motifs (Supplementary Figure S1). It is tempting to speculate whether or not these variations function in the adaptation to specific host plants and/or evolved to escape from detection by the plant immune system.

To demonstrate functional equivalence with the putative translocon protein HrpF (Büttner et al., 2002), we tested whether or not an *hpaT* mutant can be complemented by *hrpF* from *X. euvesicatoria*, and *vice versa*, whether an *hrpF* mutant can be complemented by *hpaT* from *X. translucens* strain CFBP 2541. For the assay we infiltrated pepper leaves of the cultivar ECW-10R (containing the resistance gene *Bs1*), which trigger a race-specific HR to *X. euvesicatoria* strains expressing the *avrBs1* gene and a non-host HR to some strains of *X. translucens* (e.g., UPB787 and UPB820). While the *hrpF* mutant could hardly be complemented, as indicated by the occasional presence of little brown spots in the area of infiltration, we observed strong complementation of the *hpaT* mutant by *hrpF*. This result was rather surprising given the fact that the HrpF homolog NolX, which is 48% identical to HrpF, was not able to complement an *hrpF* mutant (Huguet and Bonas, 1997). Notably, HpaT and HrpF belong to distinct protein families, which share only 16% or less amino acid sequence identity in pairwise comparisons (Supplementary Figure S8 and Table S3). From these results, we conclude that HpaT and HrpF may be analogous translocon proteins, which are to some degree functionally interchangeable.

Surprisingly, the polar insertion mutant in *hpaT* could be partially complemented by *hpaT*, but also by the downstream gene *hpaH*. This finding is puzzling since HpaH was initially predicted to act in the periplasmic space and may be involved in remodeling of the peptidoglycan layer thanks to its predicted lytic transglycosylase activity, thus helping to build up the T3SS machinery (Noël et al., 2002; Zhang et al., 2008). To that effect, an N-terminal signal peptide for the general Sec-dependent secretion pathway is predicted for HpaH using Phobius, and enzymatic assays with Hpa2 from *X. oryzae* revealed lytic activity against the bacterial cell walls (Zhang et al., 2008).

Conflicting data exist, however, with respect to the export pathway and contribution to pathogenicity in the several *Xanthomonas* strains. Mutant analyses in *hpaH* (*X. euvesicatoria*) or its homolog *hpa2* in *X. oryzae* had indicated only minor (*hpaH* from strain 85-10, *hpa2* from strain RS105) or no (*hpa2* from strain PXO99^A^) effect on the interaction with its host plants (Zhu et al., 2000; Noël et al., 2002; Li et al., 2011a). However, others have demonstrated a significant reduction in symptoms and bacterial counts on rice when *hpa2* was inactivated (strain PXO99^A^) (Zhang et al., 2008). Similarly confusing is the observation that a *hpa2* mutant in the *X. oryzae* strain PXO99^A^ completely loses its ability to cause a non-host HR on tobacco (*Nicotiana benthamiana*) (Zhang et al., 2008), while a *hpa2* mutant in the *X. oryzae* strain RS105 triggered an HR similar to the wild type (Li et al., 2011a). Since it was shown by reporter fusions that HpaH contributes to the secretion of a subset of effectors by *X. euvesicatoria* (Büttner et al., 2007), subtle differences in the experimental conditions and/or differences in the sets of affected T3Es in the various strains may explain these results, which are otherwise hardly to reconcile.

The HpaH protein has been localized to the periplasmic space and in the extracellular milieu (Hausner et al., 2017). Conficting data exist whether export of HpaH/Hpa2 depends on the T3SS or not (Li et al., 2011a; Hausner et al., 2017). Similarly, while HpaH was reported to bind to the peptidoglycan as well as to periplasmic components of the T3SS (Hausner et al., 2017). Li et al. (2011a) reported that Hpa2 has the ability to bind to the host cell membrane. These apparently conflicting data may suggest that HpaH and Hpa2, which are 89% identical to each other at the amino acid level, act in a different manner. Since HpaH from *X. translucens* is basically equally distant (35% identity) to both HpaH from *X. euvesicatoria* and Hpa2 from *X. oryzae* one cannot derive at conclusions on its mode of action based on sequence similarity.

Our results suggest that both proteins, HpaT and HpaH, contribute to the function of the T3SS. Since the *hpaT* mutant could be partially complemented by a plasmid-borne copy of *hpaH*, HpaT is not absolutely required for the translocation of effector proteins into plant cells but rather serves as a facilitator of transport. To avoid multicopy effects, it would be interesting to perform complementation assays with chromosomal insertions of *hpaT*, *hpaH*, or both genes. Similar observations as with *hpaT* have been made for the other candidate translocon gene, *hrpF* from *X. oryzae.* Mutations in *hrpF* led to drastic loss of virulence but did not completely eliminate pathogenicity (Sugio et al., 2005; Cho et al., 2008; Li et al., 2011a). This finding is also reminiscent of the phenotype of a *P. syringae* mutant in *hrpK*, which shares low similarity with *hrpF* from *X. euvesicatoria* (Petnicki-Ocwieja et al., 2005). Strikingly, while single mutants in *hrpF* and *hpa2* still provoked symptoms on rice, only a double mutant in both genes was non-pathogenic on its host (Li et al., 2011a). Moreover, it was shown by yeast two-hybrid and protein pull-down experiments that Hpa2 interacts with HrpF, and bimolecular fluorescence complementation assays using split YFP demonstrated that both proteins can interact in the plant cell membrane (Li et al., 2011a). From these results, along with data from several avirulence reporter experiments, Li et al. (2011a) concluded that both proteins, Hpa2 and HrpF, act together to translocate T3Es into plant cells, even if each protein alone has sufficient activity to allow effector translocation at much reduced rate.

Our complementation data suggest a mechanism for *X. translucens* where both HpaH and HpaT contribute to effector translocation, yet the presence of only one of the two protein is still sufficient to allow some effector delivery to the plant cell. A similar redundancy of translocon components was also found in *P. syringae* where a quintuple mutant in the candidate translocon components *hrpK1*, *hrpW1*, *hrpZ1*, *hopP1*, and *hopAK1* could be complemented by each of these genes alone except for *hopP1*, which suggested that a consortium of semi-redundant translocators cooperate in the translocon formation (Kvitko et al., 2007). A similar multi-component translocon complex could operate in *Xanthomonas*, that may involve glycine-rich proteins (e.g., XopA, syn. Hpa1) as a third component (Sugio et al., 2005). These results challenge the view of a simple, single-protein translocon pore; more work is needed to understand the molecular details at the “port of entry” (Büttner and Bonas, 2002b).

## Author Contributions

RK and CB conceived the study. RK and CP performed the bioinformatic analyses. CP, EB, and TV generated mutants and constructed plasmids. CP, EB, JJ, MP, and RK conducted plant assays. JJ, MP, and RK designed, performed and analyzed the qRT-PCR experiments. All authors analyzed the data, and wrote the manuscript. All authors have read and approved the final manuscript.

## Conflict of Interest Statement

The authors declare that the research was conducted in the absence of any commercial or financial relationships that could be construed as a potential conflict of interest.

## Footnotes

1 http://blast.ncbi.nlm.nih.gov

2 http://www.ebi.ac.uk/Tools/msa/muscle/
