# Supplementary material for: Comparative Genomics Identifies a Novel Conserved Protein, HpaT, in Proteobacterial Type III Secretion Systems that Do Not Possess the Putative Translocon Protein HrpF
Source: Front Microbiol. 2017 Jun 26;8:1177. doi: 10.3389/fmicb.2017.01177 (PMC5483457; doi:10.3389/fmicb.2017.01177)
Supplement: Supplementary file 1 [file Table_1.PDF]

**SUPPLEMENTARY TABLE S1 | Oligonucleotides used in this study.**

| Name | Sequence | Purpose |
|---|---|---|
| pVO155-Fw | 5′-GAGATCCCCAGCCCGCCTAATG | Verification of constructs in pVO155 |
| pVO155-Rev2 | 5′-GCGCGTTACAAGAAAGCCGG | Verification of constructs in pVO155 |
| pBBR-Fw | 5'-AGAAGCACACGGTCACACTG | Verification of constructs in pBBR1MCS-5 |
| pBBR-Rev3 | 5'-CCGGCTCGTATGTTGTGTGG | Verification of constructs in pBBR1MCS-5 |
| Xt-hrcT-Fw | 5'-GCAGGATCCGACCTGGCCGGTTTCAAC | Cloning of an internal *hrcT* fragment for mutagenesis |
| Xt-hrcT-Rev | 5'-CGCTCTAGAAGCAACTGCGAAACGA | Cloning of an internal *hrcT* fragment for mutagenesis |
| Xt-hrcT-cpt-Fw | 5'-CTAGGTCGACGAGAGAATCGTTATGAATGTGTCCGATGCCG | Cloning of *hrcT* in pBBR1MCS-5 for complementation |
| Xt-hrcT-cpt-Rev | 5'-CTAGGAATTCTTAGTGGGTAGCAGGAGACC | Cloning of *hrcT* in pBBR1MCS-5 for complementation |
| Xt-hpaT-Fw | 5'-GCAGGATCCACCGCCAAGGCCGAGTTC | Cloning of an internal *hpaT* fragment for mutagenesis |
| Xt-hpaT-rev | 5'-CGCTCTAGAGCATCCACAGCGGCTTGC | Cloning of an internal *hpaT* fragment for mutagenesis |
| Xt-hpaT-cpt-Fw1 | 5'-CTAGAAGCTTGAGAGAATCGTTATGAGCCTTAAAAGTTTCT | Cloning of *hpaT* in pBBR1MCS-5 for complementation |
| Xt-hpaT-cpt- Rev1 | 5'-CTAGTCTAGACTACACCGTATGGAGCTGGAA | Cloning of *hpaT* in pBBR1MCS-5 for complementation |
| Xt-hpaH-cpt-fw | 5'-CTAGAAGCTTGAGAGAATCGTTATGCGATGGACAGGATGGC | Cloning of *hpaH* in pBBR1MCS-5 for complementation |
| Xt-hpaH-cpt-rev | 5'-CTAGTCTAGATCAACACGCAGCCAGATCCT | Cloning of *hpaH* in pBBR1MCS-5 for complementation |
| Xt-hgiB-Fw | 5'-GCAGGATCCAGCCTCGCTGGCGCAGATGA | Cloning of an internal *hgiB* fragment in pVO155 |
| Xt-hgiB-Rev | 5'-CGCTCTAGAGCACCGCCTGCAGACCGGCC | Cloning of an internal *hgiB* fragment in pVO155 |
| XT_hpaT-fwd1 | 5'-CAAGCCGCTGTGGATGCT | Expression analysis of *hpaT* gene (qRT-PCR) |
| XT_hpaT-rev1 | 5'-GAGGCCAGATCCTTTTTCAGC | Expression analysis of *hpaT* gene (qRT-PCR) |
| XT_hpaT-fwd2 | 5'-TCAACCTGTGCACCTCGC | Expression analysis of *hpaT* gene (qRT-PCR) |
| XT_hpaT-rev2 | 5'-CATCCACAGCGGCTTGCG | Expression analysis of *hpaT* gene (qRT-PCR) |
| XT_hpaH-fwd1 | 5'-CAGATGATGAACAAGTACGGCA | Expression analysis of *hpaH* gene (qRT-PCR) |
| XT_hpaH-rev1 | 5'-CTGGTCCGATTCCAGGATCT | Expression analysis of *hpaH* gene (qRT-PCR) |
| XT_hpaH-fwd2 | 5'-CGCGTGCTCAAGGCCATC | Expression analysis of *hpaH* gene (qRT-PCR) |

| | | |
|---|---|---|
| XT_hpaH-rev2 | 5'-CGCCGCAGATGGATCGAA | Expression analysis of *hpaH* gene (qRT-PCR) |
| XT_hpaT-hpaH-operon-fwd | 5'-GCGGACGTGTCGTTCCAG | Expression analysis of *hpaT-hpaH* operon (qRT-PCR) |
| XT_hpaT-hpaH-operon-rev | 5'-CTCAAAGCAACCGGCATGTG | Expression analysis of *hpaT-hpaH* operon (qRT-PCR) |
| XT_hgiB-fwd | 5'-ATGAGCACGCACAAGGGTC | Expression analysis of *hgiB* gene (qRT-PCR) |
| XT_hgiB-rev | 5'-GCCCCTCTGATGTGCCG | Expression analysis of *hgiB* gene (qRT-PCR) |
| XT_hrpB1-fwd | 5'-CTGATCGAAATCATTACCGTGGC | Expression analysis of *hrpB1* gene (qRT-PCR) |
| XT_hrpB1-rev | 5'-CGCGCTTGATCGCCAGC | Expression analysis of *hrpB1* gene (qRT-PCR) |
| XT_hrpC1-fwd | 5'-TCGAACCGAAACTGGACGC | Expression analysis of *hrpC1* (*hrcU*) gene (qRT-PCR) |
| XT_hrpC1-rev | 5'-CGCCTTGACCAGCATCTTGA | Expression analysis of *hrpC1* (*hrcU*) gene (qRT-PCR) |

Restriction sites of the enzymes used for cloning are underlined.
