# Supplementary material for: Comparative Genomics Identifies a Novel Conserved Protein, HpaT, in Proteobacterial Type III Secretion Systems that Do Not Possess the Putative Translocon Protein HrpF
Source: Front Microbiol. 2017 Jun 26;8:1177. doi: 10.3389/fmicb.2017.01177 (PMC5483457; doi:10.3389/fmicb.2017.01177)
Supplement: Supplementary file 2 [file Table_2.pdf]

SUPPLEMENTARY TABLE S2 | Relative expression of putative HrpX target genes in strains of *Xanthomonas translucens*, as revealed by quantitative reverse transcription PCR (qRT-PCR).

	Target gene(s)	*hpaT*	*hpaT*	*hpaT*	*hpaH*	*hpaH*	*hpaH*	*hgiB*	*hrpB1*	*hrpC1*	*hpaT-hpaH* operon
	Primer pair	1	1	2	1	1	2	1	1	1	1
	cDNA dilution	1/5	1/10	1/10	1/5	1/10	1/10	1/10	1/10	1/10	1/10
**Strain**	**Replicate**										
**UPB820 (pBBR1MCS-5)**	1	29.77	30.69	31.85	31.06	32.99	33.82	36.10	29.08	32.34	40
	2	29.86	30.51	31.15	30.97	33.10	ND	ND	28.64	30.29	40
**UPB820 (pBBR1MCS-5::*hrpX*)**	1	21.15	22.68	21.76	24.21	24.93	23.36	23.26	20.17	22.93	32.66
	2	21.10	22.99	21.86	24.17	24.87	23.53	22.99	21.28	21.92	31.90
**UPB820 (pBBR1MCS-5::*hrpG**)**	1	19.92	21.59	20.60	23.16	24.94	21.69	21.29	18.61	22.63	29.46
	2	19.93	21.83	20.60	23.08	35.62	21.81	21.07	18.58	22.65	29.55
**UPB820 (pBBR1MCS-5)**	**Average**	29.82	30.60	31.50	31.02	33.05	33.82	36.10	28.86	31.32	40
**UPB820 (pBBR1MCS-5::*hrpX*)**	**Average**	21.13	22.84	21.81	24.19	24.90	23.45	23.13	20.73	22.43	32.28
**UPB820 (pBBR1MCS-5::*hrpG**)**	**Average**	19.93	21.71	20.60	23.12	30.28	21.75	21.18	18.60	22.64	29.51
**UPB820 (pBBR1MCS-5::*hrpX*)**	**Δ Ct**	8.69	7.77	9.69	6.83	8.15	10.38	12.98	8.14	8.89	7.72
**UPB820 (pBBR1MCS-5::*hrpG**)**	**Δ Ct**	9.89	8.89	10.90	7.90	2.77	12.07	14.92	10.27	8.68	10.50

Average: Arithmetic mean of the two technical replicates shown above
Δ Ct: Difference between the strain ectopically expressing the regulator versus a strain containing the empty vector

ND: Amplification not conclusive, indicative of absence of gene expression under this condition
40: Ct value larger than 40, indicative of absence of gene expression under this condition

Remark: The red value indicates an outlier, corresponding to the second technical replicate for the first primer pair at a 1/10 cDNA dilution
