# Supplementary material for: Comparative Genomics Identifies a Novel Conserved Protein, HpaT, in Proteobacterial Type III Secretion Systems that Do Not Possess the Putative Translocon Protein HrpF
Source: Front Microbiol. 2017 Jun 26;8:1177. doi: 10.3389/fmicb.2017.01177 (PMC5483457; doi:10.3389/fmicb.2017.01177)
Supplement: Supplementary file 3 [file Table_3.PDF]

SUPPLEMENTARY TABLE S3 | Pairwise sequence comparisons of HrpF and HpaT proteins.

	XCC_HrpF	RS_PopF1	RS_PopF2	UG_HrpF	XTC_HpaT	CF_HpaT	PA_HpaT
**XCC_HrpF**		39	35	39	13	10	12
**RS_PopF1**	53		82	53	15	12	14
**RS_PopF2**	50	86		47	16	10	14
**UG_HrpF**	52	68	62		16	11	13
**XTC_HpaT**	24	26	24	27		18	22
**CF_HpaT**	16	19	16	20	30		38
**PA_HpaT**	20	19	20	21	36	51	

Sequences were aligned with the Needleman-Wunsch algorithm for global alignments, using default parameters (https://blast.ncbi.nlm.nih.gov/). Sequence identity (blue, in %) and sequence similarity (red, in %) are tabulated. Sequences correspond to the genes shown in **Figure 2**. HrpF homologs are from *X. citri* pv. *citri* (XCC_HrpF), *R. solanacearum* (RS_PopF1 and RS_PopF2) and *U. gangwonense* (UG_HrpF). HpaT homologs are from *X. translucens* pv. *cerealis* (XTC_HpaT), *C. fungivorans* (CF_HpaT) and *P. andropogonis* (PA_HpaT).
