# Supplementary material for: Comparative Genomics Identifies a Novel Conserved Protein, HpaT, in Proteobacterial Type III Secretion Systems that Do Not Possess the Putative Translocon Protein HrpF
Source: Front Microbiol. 2017 Jun 26;8:1177. doi: 10.3389/fmicb.2017.01177 (PMC5483457; doi:10.3389/fmicb.2017.01177)
Supplement: Supplementary file 6 [file Image_3.PDF]

hpaT

```
XH_CFBP1156    CGAAGCCGGTCCGCCGCTTGCGAAGCGAACGCA---------CCGATCGGGCGGCCCGATCGGCGCGTTCGCGCTTGTTTCGCAGATGGAGCGATCGATTCGTTGGC-GTGGCTGCGCTA
XT_CFBP4691    -----CTGGACCGCCGCCAGCGAAGAAACCGCAGCGCTGGCGGCGAGCAATCGATGCG--C---TCGCTTG---TTTCTTCGCGGATGACGCGATTGGTTCGTAAGCCGCCGCCGCCGCG
XTP_B99        -------CGACCGCCTTGAGCGAAGAAGAATCG----TGCCGCTGGGC-GTCGATGTG--CGGATCTTTTGGCCTTGTTTCGCAGAGAGAGCGATCGGTTCGTTGGCAGTGGCCGGCGCG
XTG_CFBP2053   -------CGACCGCCTTGAGCGAAGAAGAATCG----TGCCGCTGGGC-GTCGATGTG--CGGATCTTTTGGCCTTGTTTCGCAGAGAGAGCGATCGGTTCGTTGGCAGTGGCCGGCGCG
XTC_CFBP2541   -------GGACCGCCGTGAGCGAAGAAAGAGCG----TGCCAGCGGG--GGTGATGCG--CGGATCTTTTCGCCTTGTTTCGCAGAGGGAGCGATCGGTTCGGTTGAAGCGGCAAGTGTA
XTU_Xtu4699    -------GGACCGCCGTGAGCGAAGAAAGATCG----TGCCACTGGGC-ATCGATGCG--CGGATCTTTCAGCCTTGTTTCGCAGAGGGAGCGATTGGTTCGGTTGCAGCGGCCAGCGCA
XTT_DSM18974   ------CGGACCGCCGTGAGCGAAGAAAGAGCG----TGCCAGTGGG--GGCGATGCG--CTGATCTTTTCGCCTTGTTTCGCAGAGGGAGCGATTGGTTCGGTTGCAGCCGCTAGCGCA
                       * *****     ******      *            *       *    *  *     *  *     **  ***** **    ***** * ****    *  *  **

XH_CFBP1156    GGTGCGGATCGGTATA-TGTTGGACTGCAGCGGCGCATC--GCCGCAGTTC-GCGGGCCGCTACAGGTCTGCTCCAACAATC---GAGGAGTTTTTT
XT_CFBP4691    CGCGTGCATCGTCCTA-TGTTGGACGACAGCGGCGTAGTCGACCGCAACGCCGCGGACCGCGACCGGTCCGCCCCAACATGC---GAGGACGTTGCG
XTP_B99        CGCGCGCATCGTCTTAATGTTGGATTGCGGCCGTCTGGTCGGTCGCTGGTC--CGGACCGGCACCGGTTCACTCCGACAAACAA-GAGGACTTTGCG
XTG_CFBP2053   CGCGCGCATCGTCTTAATGTTGGATTGCGGCCGTCTGGTCGGTCGCTGGTC--CGGACCGGCACCGGTTCACTCCGACAAACAA-GAGGACTTTGCG
XTC_CFBP2541   CGCGCGCCTGCACTTAGTGTTGGATCGCGGCCGTCTGATCGGTCGCTGGTC--CGGGCCGGAACCGGTTCACTTCGACAAAAAATGAGGACTTTTCG
XTU_Xtu4699    CGCGTGCGTGGGCTTAGTGTTCGATCGCGGCCGTCTGGTCGGTCGCTGGGC--CGGGCCGGCACCGGTTCACTCCGACAAACAA-GAGGACTTTGCG
XTT_DSM18974   CGCGTGCCTGGACTTAGTGTTTGATCGCGGCCGTCTGGTCGGTCGCTGGGC--CGGGCAGGCACCGGTTCACTCCGACAAAAAA-GAGGACTTTTCG
                * * *  *     ** **** **    * ** *          ***     *   *** * *  ** ***    *  * ***      *****  **
```

hgiB

```
XT_CFBP4691    -----------GGCGAGT-----TCCCGGGCG-----GACA-GGCGA-------ACCCCTGCCTCGGCGCGCCCGGCAGGCGTGGATCTCGGCAAGCACCCCACTGCCTCGTCCCGAGAT
XTT_DSM18974   ---CGGCGCATGGCGGGCCAGCCTCGCTGGCGCAGATGACATGACG------------ATGACGTGGCGGTGCGGGCCGGCCCTGCCGCCCGCAAG---CCCGCTTGCCGGTCGC-----
XTP_B99        ------------GCGGGCCAGCCTCGCTGGCGCAGATGACATGACGATGACGTGCGCCATGACGTGGTGGTGCGGGCCGGTCCGGCCGCCCGCAAG---CCTGCTGGCCAGTCGC-----
XTC_CFBP2541   GTTCGGCGCACGGTGGGCCAGCCTCGCTGGCGCAGATGACATGACG------------ATGACGTGGCGGTGCGGGCCGGCCCCGCCGTCCGCAAG---CCCGCTGGCCTGTCGC-----
XTU_Xtu4699    ---CGGCGCACGGCGGGTCAGCCTCGCTGGCGCAGATGACATGACG------------ATGACGTGGCGGTGCGGGCCGGCCCTGCCGCCTGCAAG---CCCGCTCGCCGGTCGC-----
XTG_CFBP2053   ---CGGCGCACGGCGGGCCAGCCTCGCTGGCGCAGATGACATGACG------------ATGACGTGGCGGTGCGGGCCGGCTATGCCGCCCGCAAG---CCCGCTGGCCGGTCGC-----
                           *  *  *      ** * ****      **** * **              ** *  ** *   * *** **     *    * *****    **   **   *  *** *

XT_CFBP4691    CGCCGCCGGTTTCGCGCTGCTTTCCGAAGCTTCGTGTTTCATGGCGCGGGCGATGTCAAGCTGCTTGACGATCGACTTTCCGTTGTGATGTCCACGAAGGAGAAAACAA
XTT_DSM18974   -----CTGTTTTCGGTTTGCCTTCCGAAAATTCGTGGTTCATGGCCCGCGCGATTTCACGCCGATCCAAAATGGATGCCTTGTTTCGATCTACACGAAGGAAGCAATC-
XTP_B99        -----CTGTATTCGCTTCGCTTTCCGAAAATTCGTGGTTCATGGCCCGCGCGGTTTCACGCCGATCCAAAATGGACGCCCTGTTCCGATTTATACGAAGGAAACA----
XTC_CFBP2541   -----CTGTTTTCGGTTTGCTTTTCGGAAATTCGTGGTTCATGGCCCGCGCGGTTTCACGCCGATCCAAAATGGACGTCCTGTTTCGATTTCTACGAAGGAGACA----
XTU_Xtu4699    -----CTGTATTCGGTTTGCTTTCCGGAAATTCGTGGTTCATGGCGCGCGCGGTTTCACGCCGATCCAAAATGGATGTCCTGTTTCGATTTCTACGAAGGAAACAATG-
XTG_CFBP2053   -----CTGTATTCGTTTTGCTTTCCGGAAATTCGTGGTTCATGGCCCGCGCGGTTTCACGCCGATCAAAAATGGACGTCCTGTTTCGATTTCTACGAAGGAAACAATC-
                     * *  ****    ** ** ** *  ****** ******** ** *** * *** ** * *  *  ** **      ***  *** *  ********    *
```

SUPPLEMENTARY FIGURE S3 | Comparison of *hpaT* and *hgiB* promoter regions proteins from clade-1 xanthomonads.

Promoter regions encompassing 200 bp upstream of the predicted translational start codon were aligned with the Multiple Sequence Comparison by Log-Expectation (MUSCLE) algorithm, using default parameters (http://www.ebi.ac.uk/Tools/msa/muscle/). Sequences correspond to the genes shown in **Figure 1**. PIP half boxes are shown in blue, −10 promoter motifs are shown in orange and Shine-Dalgarno sequences are shown in green. Deviations from the consensus sequence of the −10 motif are highlighted in yellow.
