# Supplementary material for: Comparative Genomics Identifies a Novel Conserved Protein, HpaT, in Proteobacterial Type III Secretion Systems that Do Not Possess the Putative Translocon Protein HrpF
Source: Front Microbiol. 2017 Jun 26;8:1177. doi: 10.3389/fmicb.2017.01177 (PMC5483457; doi:10.3389/fmicb.2017.01177)
Supplement: Supplementary file 7 [file Image_4.PDF]

hpaT-hpaH region

```
XTP_B99       GCTTGCTTTCCGCCT-CGGCGGAC------GTGTCCTTCCAGTTCGGTGCGGTGTAGCCCGTATG---CGACGGATAGGATGGCTGCTTGCGGCGTTACTAGCCACGCCGCTGGC
XTG_CFBP2053  GCTTGCTTTCCGCCT-CGGCGGAC------GTGTCGTTCCAGTTCGGTGCGGTGTAGCCCGTATG---CGACGGATAGGATGGCTGCTTGCGGCGTTACTAGCCACGCCGCTGGC
XTU_Xtu4699   CGGCGCTTTCCGCCG-CGGCGGAC------GTGTCGTTCCAGCTCCGTGCGGTGTAGCCCGTATG---CGACGGATAGGATGGCTGCTTGCGGCATGGCTAGCCACGCCGCTGGC
XTC_CFBP2541  CGGCGCTTTCCGCCT-CGGCGGAC------GTGTCGTTCCAGCTCCATACGGTGTAGCCCGTATG---CGATGGACAGGATGGCTGCTTGCGGCGTGGCTAGCCACGCCGCTGGC
XTT_DSM18974  CGGCGCTTTCCGCCT-CGGCGGAC------GTGTCGTTCCAGCTCCATACGGTGTAGCCCGTATG---CGACGGATAGGATGGCTGCTTGCGGCGTGGCTAGCCACGCCGCTGGC
XH_CFBP1156   CAGCG---TACGCCA-CGGCGACCCCGCGGGTGGCGACGCAGTTGT---CGGCGTAG-CCTCATGCGTCGTCGTGCCGGAGCGTTGCTTGCGGCGTGGCTCGCCACGCCGTT---
XT_CFBP4691   CCTCGTTGTCCGTCGCCGGCGGC-------GGTTCGGCGGCGTTGCATAGGGCGTAG-CCGCATG---CGTGGCGCAGTCGTCTTGCTGGTGGTCTGGTTCGCCACGCCGCTGGC
                  *   * ** *  *****         *   *      * *      ** **** **  ***   **  *    *      **** * **  *   * ********* *
```

SUPPLEMENTARY FIGURE S4 | Comparison of the intergenic region between *hpaT* and *hpaH* from clade-1 xanthomonads.

Gene regions encompassing the last 50 bp of the *hpaT* coding sequences, the downstream intergenic regions, and the first 50 bp of the *hpaH* coding sequences were aligned with the Multiple Sequence Comparison by Log-Expectation (MUSCLE) algorithm, using default parameters (http://www.ebi.ac.uk/Tools/msa/muscle/). Sequences correspond to the genes shown in **Figure 1**. Stop codons are highlighted in red and predicted start codons are highlighted in green.
