# Supplementary material for: Comparative Genomics Identifies a Novel Conserved Protein, HpaT, in Proteobacterial Type III Secretion Systems that Do Not Possess the Putative Translocon Protein HrpF
Source: Front Microbiol. 2017 Jun 26;8:1177. doi: 10.3389/fmicb.2017.01177 (PMC5483457; doi:10.3389/fmicb.2017.01177)
Supplement: Supplementary file 8 [file Image_5.PDF]

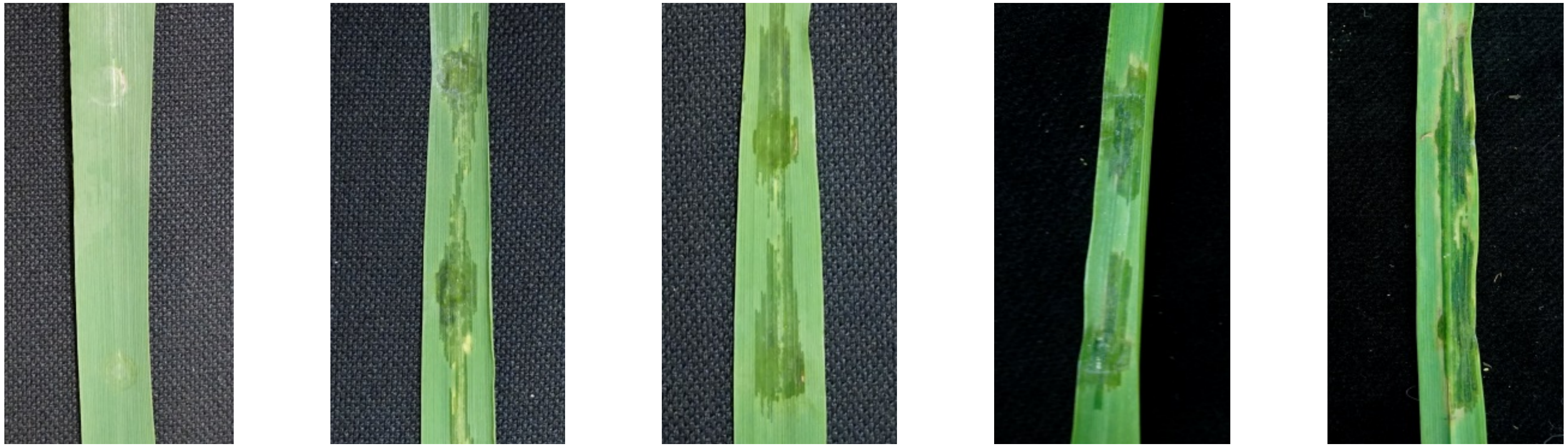

Water | UPB787^R | UPB787^R *hgiB*$^-$ | UPB820^R | UPB820^R *hgiB*$^-$

SUPPLEMENTARY FIGURE S5 | The *hgiB* gene is not required for pathogenicity of *X. translucens* on barley.

Barley leaves were infiltrated with aqueous suspensions (OD_{600}=0.5) of the wild-type strains UPB787^R and UPB820^R, and with their *hgiB* mutants. Infiltrations with water served as negative control. Symptoms were assessed one week after infiltration.
