# Supplementary material for: Comparative Genomics Identifies a Novel Conserved Protein, HpaT, in Proteobacterial Type III Secretion Systems that Do Not Possess the Putative Translocon Protein HrpF
Source: Front Microbiol. 2017 Jun 26;8:1177. doi: 10.3389/fmicb.2017.01177 (PMC5483457; doi:10.3389/fmicb.2017.01177)
Supplement: Supplementary file 9 [file Image_6.pdf]

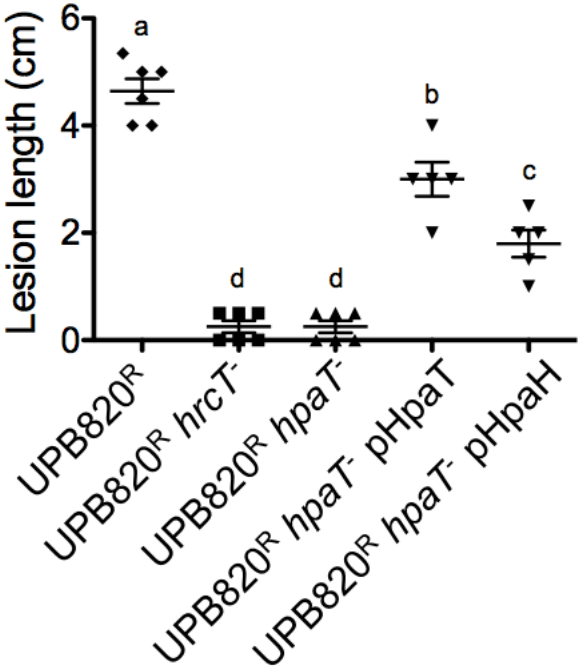

SUPPLEMENTARY FIGURE S6. The *hpaT* mutant in *X. translucens* strain UPB820^R is non-pathogenic on barley. Barley leaves were inoculated with the wild-type strain UPB820^R, the *hrcT* mutant (UPB820^R *hrcT*$^-$), the *hpaT* mutant (UPB820^R *hpaT*$^-$), the *hpaT* mutant complemented with the *hpaT* gene (UPB820^R *hpaT*$^-$ pHpaT) or with the *hpaH* gene (UPB820^R *hpaT*$^-$ pHpaH). Lesion length was measured at 15 dpi. At least five plants were used per treatment. Lower case letter represents statistically significantly different treatments based on a Students t-test (p<0.01). Treatments with the same letters are not statistically significantly different. Error bars represent the standard error of the mean.
