# Supplementary material for: Comparative Genomics Identifies a Novel Conserved Protein, HpaT, in Proteobacterial Type III Secretion Systems that Do Not Possess the Putative Translocon Protein HrpF
Source: Front Microbiol. 2017 Jun 26;8:1177. doi: 10.3389/fmicb.2017.01177 (PMC5483457; doi:10.3389/fmicb.2017.01177)
Supplement: Supplementary file 10 [file Image_7.PDF]

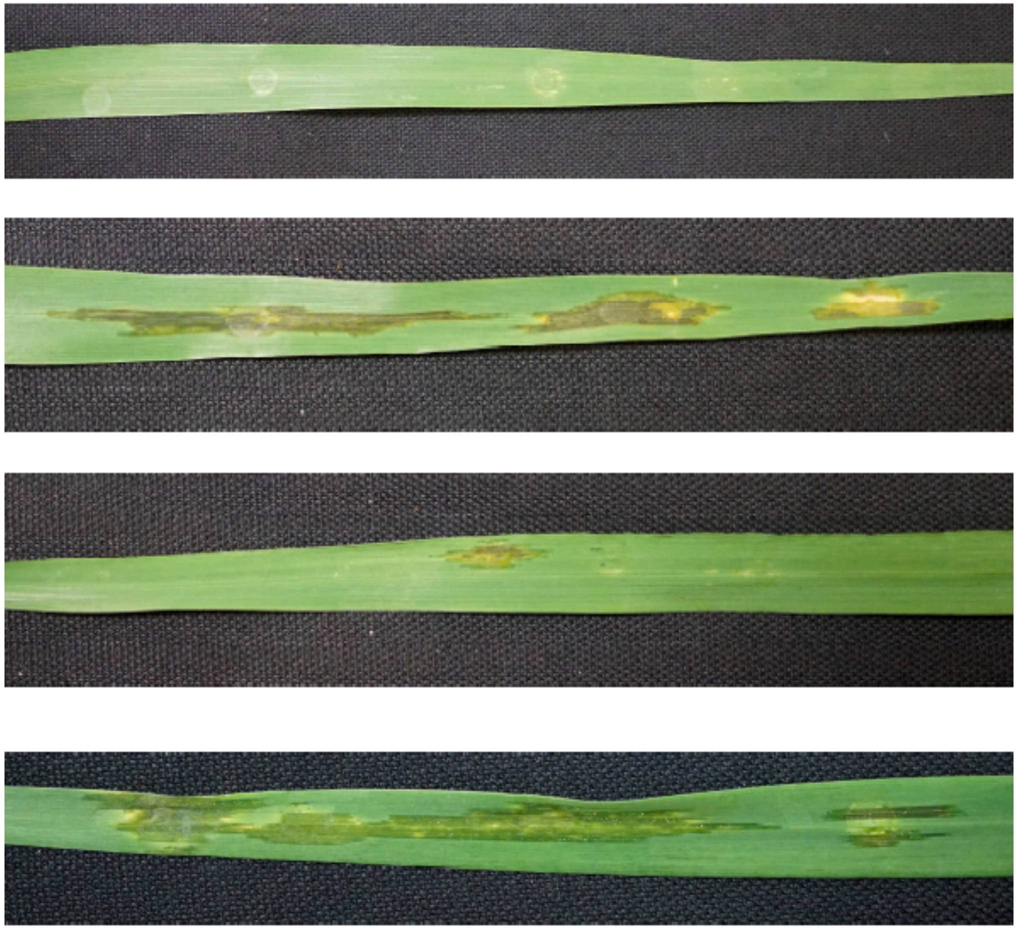

Water

UPB820^{R}

UPB820^{R} *hpaT*$^{-}$

UPB820^{R} *hpaT*$^{-}$ pHpaT

SUPPLEMENTARY FIGURE S7 | The *hpaT* mutant in *X. translucens* strain UPB820^{R} is non-pathogenic on barley.

Barley leaves were infiltrated with aqueous suspensions (OD_{600}=0.5) of the wild-type strain UPB820^{R}, the *hpaT* mutant (UPB820^{R} *hpaT*$^{-}$) and the *hpaT* mutant complemented with the *hpaT* gene (UPB820^{R} *hpaT*$^{-}$ pHpaT). Infiltrations with water served as negative control. Symptoms were assessed one week after infiltration.
