# Supplementary material for: Comparative Genomics Identifies a Novel Conserved Protein, HpaT, in Proteobacterial Type III Secretion Systems that Do Not Possess the Putative Translocon Protein HrpF
Source: Front Microbiol. 2017 Jun 26;8:1177. doi: 10.3389/fmicb.2017.01177 (PMC5483457; doi:10.3389/fmicb.2017.01177)
Supplement: Supplementary file 11 [file Image_8.PDF]

```
XCC_HrpF        MGAPVRPYKRKLIRRCFCQFCHCEAAHMSLNTLSTGSNPS-QLLGTPLNES--DSSELLG
UG_HrpF         --------------------------MMNTTITSTTGLSYQPVMGGDSESSGTDNSATAK
RS_PopF1        ----------------------------MSTNISSAASPTLPLAGPGVNGPAEGKSDMPG
RS_PopF2        ----------------------------MSTNISGAASPTLPSAGAGANGPVANPPDLPS
XTC_HpaT        ----------------------------------MSLKSFFKSIGHGIT----NAVEDVA
CF_HpaT         ------------------------------MSIFSKIKHAFEKVGHDIE----HVAKAAI
PA_HpaT         ------------------------------MSIFSKIKHAFQHLGHDIE----NAAKAVV
                                                            *

XCC_HrpF        SDSSKDDSDLPSTMDDIFLLIYQLLAALQTNAPTSTSGNGSADAPTSAPSGDASTPSSAS
UG_HrpF         TVVDDANSPLTQAINRLVQDSLQQQDSTNSN----TSGSYTTDDSSADSSTDAGTQATAT
RS_PopF1        SLFFQFDHSTGSTRPDLPTDLFFSFGDSISRAVQDASNQSPEQAPAADPA-----PASPD
RS_PopF2        SLLFQFDHPTGPSRPDLPPELFFKLDESIARAMQGAAQQSPD--PSAGPESQGNQPAPVD
XTC_HpaT        HGVEQGAKDIAKGVEGAVEHGVHAFGDVLQGDFRGGLHELGQAGKAVGDTAKAEFKLGLT
CF_HpaT         HDVEGVVKTMEKDVEHVLQDTMKMTEALMQGNFKEGLQDFLKVTDDVVKSGTDLATSQAK
PA_HpaT         HGVEHVVHALENDIKKVLQDALAMTEALMQGNLKEAMQDLLKTAEDVEKTASDAYTAGAE
                                                      .

XCC_HrpF        DDQANQPIEKRTSWPSLGYDFDPKDIKGKDAPPALEGSTVTWNGGTLTPSELQIVSTLNA
UG_HrpF         ---ADTPESSAPSAD------------------------LTWNSGTLTQKELKIVSVLNL
RS_PopF1        ---GQSCQPSQPATPPVGSD-------------------VTWNGGTLNDTQLQIIGILNL
RS_PopF2        ---AAPPQESRPADPPLGSD-------------------VTETGGTPSPLQLQILGTLSL
XTC_HpaT        ------------------------------------------VGGAMTDLALGTVADAHL
CF_HpaT         -------------------------------------------------IAMSMMGDLHI
PA_HpaT         -------------------------------------------------ATLEGLKGLHL
                                                                   :  :

XCC_HrpF        HKDQ-MPLEYKN-------LDDKINDPSTPADLKAALQGLKQDPRLFFAIGSQGDGKCGG
UG_HrpF         YKSQTGDISWND-------LQDRINNPDTPADLREALQGLSQDWTLFSAIGSQGDGKCGG
RS_PopF1        HKDK-GDISWDK-------LQDKINDPDTPPDLKWALQALSQDFNLFQAIGSQGDGRFGG
RS_PopF2        YLNA-RGLPFGERVMTRQSLERAANNADAPADLRAAAQAMLSDPALYQAIGGN-DGKFA-
XTC_HpaT        ------------------------------------------------------------
CF_HpaT         ------------------------------------------------------------
PA_HpaT         ------------------------------------------------------------


XCC_HrpF        KIKAQDLWDFSDSHPQVKAMGGKNDQFNPKDIKGSTPPPAVDGSSVTWDGGTLTQNELEI
UG_HrpF         RISSKDLMKFSNSHSQI----------------------------LTWNGGSLNNAQLEI
RS_PopF1        KIKGKDLAEFAKSHSQV----------------------------LTWNSGTLNDSQLEI
RS_PopF2        ---RKDIDKFAKSHPQV----------------------------LTRNSGTLTDSQLEI
XTC_HpaT        ---SKGLTKLAETAKGG-------------------------------------------
CF_HpaT         ---SKGFDKFVSGAEKE-------------------------------------------
PA_HpaT         ---GKGFDKVLNTAEKG-------------------------------------------
                    :.: .. .

XCC_HrpF        VSTLNRHKDQCPLKWSDLDAKINDPSTPPDLKKALTDLQSDPRLFFAIGSQGDGK---CG
UG_HrpF         VAILDRHKDKCPISWDSIRDQANDTSNPPDLRAALQKLANDPALFYAIGSQDDGK---CG
RS_PopF1        MSILARHKDKMPVDWSSIQDKINDPSTPSDLKAALQALANDPALFFAIGSQGDGN---CK
RS_PopF2        MSILARHKDQLPVGWQSIQDKINDPSTPPDLKAALQVLANDPALFSALGSPGKGHKHKAS
XTC_HpaT        ---LDKARDAVDKSLNQVVD-------------------------SAEGAVAGGM--RCV
CF_HpaT         ---LKTVTDDVEKGVDSTVKTLADSTVG-----------------IATGSVQMLK-----
PA_HpaT         ---VETVKKDINQGIDSVASDLISSAEG-----------------IVSGSVNAVK-----
                   :    .      ..                             . *:

XCC_HrpF        G--KIKDGDLGKFVSHHAQVAEYADKQAKNYTQNYVASDSPDKTKASVMNESDAMRELYR
UG_HrpF         G--KITDKDLGKFSNNHSQVKEFNEKQAQNYMENYIPSDASQNQSASVMTENDALRELYR
RS_PopF1        G--KIKAGDVSKFADNHPQVEEYNRKKAEGYVKNYIPSDAKPGDKPSAMTQNDALRELYR
RS_PopF2        GDYRITAGDVSRFIDKHPQVEEYNRKKAEGYVKNYIPSDAKPGDKPSAMTQNDALRELYR
XTC_HpaT        D--DVAHGRFDRLGKDSLSVAG----NAFNLCTSLTPEGMVANVGANLTRSFMDGTPLSK
CF_HpaT         ---DLAHGDLKAAMGDAMKVGE----DLVTVAADLTPEGLAVDMASQVM-----------
PA_HpaT         ---DLAHGNFKGMMNDMENVAS----DALDVAADLTPEGLGANVVASTL-----------
                    :    .        .*      .      .  ...      ..

XCC_HrpF        YSDYLPDDLNQDTFKSLVDGESSTKKCPPQVIAAAQYFRDHPDQWKAL---AGDSGSMSK
UG_HrpF         YSDYLPKKLNEDTFKRIVDGTENVGKCPPQVIAAAQYFLQHRQAWSQLNQTSDDNPTMSK
RS_PopF1        YSDYLPKKLDMEAFQRIVDGDSDVKKAPPQVIAAAEYFLQNRNEWASLNKMDDPDKRVGK
RS_PopF2        YSDYLPKKLDMEAFQRIVDGDSDVKKAPPQVIAAAEYFLQNRNEWASLNKMDDPDKRVGK
XTC_HpaT        YANVIGDTMARKPLWMLRDGAKDV----------AEPFLDPLKK-----DLASADAAVNR
CF_HpaT         -----------------------------------------------------VAAHIGN
PA_HpaT         -----------------------------------------------------AAAHVGS
                                                                         :.
```

```
XCC_HrpF        PDFLQKASSEIHLTADEQKTLDTINSHQDAFYGDGKEVTRDKLDSISKDDKADPAVKEAA
UG_HrpF         SDFLHTASSSIHLNKDEIKTASTINKNLDAFFGDG-DLTRDKLASIATNKSLSNEVRDTA
RS_PopF1        SDFLQRAASAVHLSKEDLQTVSTINSNLDVFFKDGQKITRDRLAAMSQDESLSPAVRNAA
RS_PopF2        SDFLQRAASAVHLSKEDLQTVSTINSNLDVFFKDGQKITRDRLAAMSQDESLSPAVRNAA
XTC_HpaT        T--LDKAENGISGTLD------------------------------KAENGISRTLAEAA
CF_HpaT         AKIDDLVAGAMHGNVG--------------------------------------------
PA_HpaT         AQLDDAIAGAMHGGVG--------------------------------------------
                .       . :

XCC_HrpF        TQLLNDPLLFGLLNNSITGYKKPHRFFG-GGHVVDSGKISQDDFRRFYDNMSA--ANKTV
UG_HrpF         KQLLQDPLLFGLINNARSGYKTKKGFFDFGGPTVDSGVIGKKDFQQFFNNMSP--ANKSP
RS_PopF1        KQLLQDPLLYGLINNANSGYKTKNGFFSFGGPTVDSGVIGKKDFEKFMSSMTD--ANKTV
RS_PopF2        KQLLQDPLLYGLINNANSGYKTKNGFFSFGGPTVDSGVIGKKDFEKFMSSMTD--ANKTV
XTC_HpaT        VA---------------------------SGLAVQGGMTAQSQTPQLSEAQEDAIARQLA
CF_HpaT         ------------------------------------------------------------
PA_HpaT         ---------------------------------------------------------KVV


XCC_HrpF        DKPVTHDASSAEQQKAVSDMLMGKDDPPAIKKPKKDVGVFQEGLHEFLKWDSKILDWMSV
UG_HrpF         ESRVTHKTKSAESQSAVADMGMGIDDQPDIKAVKKSGGFLMHAMDKILNIAGKVFDIGSQ
RS_PopF1        QARKTHPANSEASKSAVADMGMGMEDQPDIKAVKKSGGALKKAMDKILTIYSKVMDIASQ
RS_PopF2        QARKTHPANSEASKSAVADMGMGMEDQPDIKAVKKSGGALKKAMDKILTIYSKVMDIASQ
XTC_HpaT        DAMERKDTQSGGQDNLSTQDGRGDTPLLSEAQEDAIAGQLADALNDNLS-----------
CF_HpaT         ------EMVKGAEKFAKSEVVSKVEDYAETK-----------------------------
PA_HpaT         KAVAKTEASEGAQK--LSETVLGDNGTQTAEAGATAGAALFAG---------------GA
                         .  ..   ::

XCC_HrpF        GLSALNGIPIIGEITDAASIALESEAQAAQVVDTAIQGGDMSLALKLAGINMAGAVVGAV
UG_HrpF         IVGALGFIPGFGEIADALSLVAEAESKACKILSTIIEGGNIGKAFAEAGIDMASAALGCI
RS_PopF1        VVGALGVIPGLGEIADALSMGMAAGASAAKVLSTLLNGGSLKKALAEAGINLASAALGAV
RS_PopF2        VVGALGMIPGLGEIADALSMGMAAGASAAKVLSTLLNGGSLKKALAEAGINLASAALGAV
XTC_HpaT        TQDGRGDTPQLSEAQEDAIAGQLADALNDNLSTQDGRGDTPQLSEAQED-----------
CF_HpaT         ----LASNPKIAEL-------------ASNVLPFLLLAP---------------------
PA_HpaT         GRGGRRGTQGTREPSERFAKTSSNGTKNSNKADGTSEKE---------------------
                            *                :

XCC_HrpF        GGPTARIGAKAVAKGVAEAAAKGTAK-GVAKGAGRTAAERPSAAEFAKGYVGGTAISKSV
UG_HrpF         GGPEMRM---AMREGLARKLMEKTMNTGIDMAIDQAKSFTDSYLQNLKGRLDTPSTNYLA
RS_PopF1        AGPEARV---ALKNGLTKMLVEKVANTGIDLAVDKAKSFVDGYLQDLKGRLQATAANAAN
RS_PopF2        AGPEARV---ALKNGLTKMLVEKVANTGIDLAVDKAKSFVDGYLQDLKGRLHATAANAVN
XTC_HpaT        ----------AIARQLADAMEGKDKQSGREEGL-------------LASLFGEPALSASA
CF_HpaT         ----------DVSRNQKNADNERSSHQKHAFA----------------------------
PA_HpaT         ----------ADAKQTQDKSKEPDDKTKQAAG---------------------TPPSTLN
                                         :     .

XCC_HrpF        EILKTPVLAGLHYEEYRLDKQKEGEIHQKMENAGGIPMGKQIVPTNVANNFEGDLRQNLR
UG_HrpF         TAIPA-------------------------------------------------------
RS_PopF1        TVNTSVNWVSDKTKDFLENPVQNLTPRVNIPGITPYQPGYPMVAAAA-------------
RS_PopF2        TVNTGVNWVSDKTKDFLENPVQNLTPRVNIPGITPYQPGYPVVAPAA-------------
XTC_HpaT        DVSFQLHTV---------------------------------------------------
CF_HpaT         ------------------------------------------------------------
PA_HpaT         TLLLS----------YLEGNQRKLA-----------------------------------


XCC_HrpF        NIRVRRR
UG_HrpF         -------
RS_PopF1        -------
RS_PopF2        -------
XTC_HpaT        -------
CF_HpaT         -------
PA_HpaT         -------
```

SUPPLEMENTARY FIGURE S5 | Multiple sequence alignment of HrpF and HpaT proteins.

Sequences were aligned with the MUltiple Sequence Comparison by Log-Expectation (MUSCLE) algorithm, using default parameters (http://www.ebi.ac.uk/Tools/msa/muscle/). Sequences correspond to the genes shown in **Figure 2**. HrpF homologs are from *X. citri* pv. *citri* (XCC_HrpF), *R. solanacearum* (RS_PopF1 and RS_PopF2) and *U. gangwonense* (UG_HrpF). HpaT homologs are from *X. translucens* pv. *cerealis* (XTC_HpaT), *C. fungivorans* (CF_HpaT) and *P. andropogonis* (PA_HpaT).
