# Supplementary material for: Comparative Genomics Identifies a Novel Conserved Protein, HpaT, in Proteobacterial Type III Secretion Systems that Do Not Possess the Putative Translocon Protein HrpF
Source: Front Microbiol. 2017 Jun 26;8:1177. doi: 10.3389/fmicb.2017.01177 (PMC5483457; doi:10.3389/fmicb.2017.01177)
Supplement: Supplementary file 4 [file Image_1.PDF]

A

>*Xanthomonas translucens* strain DAR61454
MSFKSFFKSVGHGIAHAAEDVAHGVEQGAKDVAKGVEGVVEHGVNAVGDVMQGDFKGSLHELGQAGKAVGDTAKAEFKLGLAVGGAMTDLALGTVAD
AHLSKGLTKLAQSAKRGMDKARDAVDKSVDQVVDSAEGVVAGGARFADDAVHGRFDRLGKDSLSVASNAFNVCTSLTPEGMAANVGANLTRSFMDGT
SLSKYADLVGDTMARKPLWMLRDGAKDVAEPLLDPLKKDLASADAAVDRTLDKAENGISRTLAEAAVASGLAVQGDMTAQS
QTPPLSEAQEDAIARQLADAMERKDEQSGGQVNLSTQNGSG
DTPLLSEAQEDAIARQLADAMEGKDKQSGREEGLLASLFGEPALSASADVSFQLRAV

>*Xanthomonas translucens* pv. *translucens* strain DSM 18974
MSLKSFFKSIGHGITNAVEDVAHGVEQGAKDIAKGVEGAVEHGAHALGDVMQGDFRGGLHELGQAGKAVGDTAKAEFKLGLTVGGAMTDLALGTVAD
AHLSKGLTKLAETAKGGLDKARDAVDKSLNQVVDSAEGAVAGGVRCVDDVAHGRFDRLGKDSLSVAGNAFNLCTSLTPEGMAANVGANLTRSFMDGT
SLSKYADVVGDTMARKPLWMLRDGAKDVAEPLLDPLKKDLASADAAVDRTLDKAEDGISRTSTEAAVASGLAVQGDMTAQS
QTPLLSEAQEDAIAGQLADALQGNLSTRDGRG
DTSLLSDAQEDAIAGQLADALQGNLSTQDGRG
DTPQLSEAQEDAIARQLADAMEGKDKQSGREEGLLASLFGEPALSASADVSFQLHTV

>*Xanthomonas translucens* pv. *cerealis* strain CFBP 2541
MSLKSFFKSIGHGITNAVEDVAHGVEQGAKDIAKGVEGAVEHGVHAFGDVLQGDFRGGLHELGQAGKAVGDTAKAEFKLGLTVGGAMTDLALGTVAD
AHLSKGLTKLAETAKGGLDKARDAVDKSLNQVVDSAEGAVAGGMRCVDDVAHGRFDRLGKDSLSVAGNAFNLCTSLTPEGMVANVGANLTRSFMDGT
PLSKYANVIGDTMARKPLWMLRDGAKDVAEPFLDPLKKDLASADAAVNRTLDKAENGISGTLDKAENGISRTLAEAAVASGLAVQGGMTAQS
QTPQLSEAQEDAIARQLADAMERKDTQSGGQDNLSTQDGRG
DTPLLSEAQEDAIAGQLADALNDNLSTQDGRG
DTPQLSEAQEDAIAGQLADALNDNLSTQDGRG
DTPQLSEAQEDAIARQLADAMEGKDKQSGREEGLLASLFGEPALSASADVSFQLHTV

>*Xanthomonas translucens* pv. *graminis* strain ART-Xtg29
MSFKSFIKSIGHGITHAAEDVAHGIEQGVKDVAKGVEGVAEHGAQALGDVMQGDFRGGLKELGQAGKSIGDTAKAEFKMGLTVGGAMTDLALGTVAD
AHLNKGLTKLAQSAKGVMDKTRDAVDKSVNQFVDSAEGAVAGGVRCAEDVAHGRLDRLGKDSLSVASNAFNVCTSLTPEGMVASVGANLTRTFLSDT
PVGKYANVVGDTMARKPLWMLRDSAKDVAEPLLEPLKKDLASADASVDRTLDKAEAGVSRTLDKAEAGVSRTLAEAAEQA
QTASLSGAIEYAIARELEKSLQPDDTQSRGLGDLSIQIGLG
DTGSLSGAMEDAIARELEQSLQPDDTQSRGLGDLSIQIGLG
DTGSLSGAMEDAIARELEQSLQPDDTQSRGLGDLSIQIGLG
DTPSLSGATEDAIARQLADALEGKDKQSTQDGRG
DTPLLSDAQADSIAGQLYDALQDNLSTQDGSG
DTPPLSEAQEDSIADQLADDLQDNLATQGGSG
DMPPLSEAQQEAIQRQLDDAMERKARQSGRDEGLLSASADVSFQFGAV

>*Xanthomonas translucens* pv. *undulosa* strain Xtu4699
MSFKSFFKSVGHGIAHAAEDVAHGVEQGAKDVAKGVEGVVEHGVNAVGDVMQGDFKGSLHELGQAGKAVGDTAKAEFKLGLAVGGAMTDLALGTVAD
AHLSKGLTKLAQSAKRGMDKARDAVDKSVDQVVDSAEGVVAGGARFADDAVHGRFDRLGKDSLSVASNAFNVCTSLTPEGMAANVGANLTRSFMDGT
SLSKYADVVGDTMARKPLWMLRDGAKDVAEPLLDPLKKDLASADAAVDRTLDKAENGISRTLAEAAVASGLAVQGDMTAQS
QTPPLSEAQEDAIARQLADAMERKDKQSTQDGRG
DTPLLSEAQEDSIAGQLADALQDNLSTQDGRG
DTPLLSEAQEDSIAGQLADALQGNLSTQDGSG
DTPPLSEAQEDAIARQLADAMERKDEQSGGQVNLSTQNGSG
DTPLLSEAQEDAIARQLADAMEGKDKQSGREEGLLASLFGEPALSAAADVSFQLRAV

B

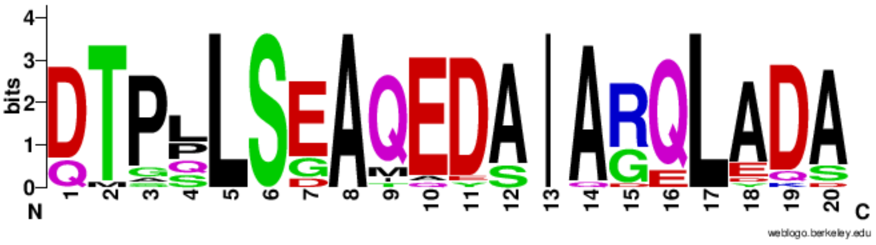

SUPPLEMENTARY FIGURE S1 | Repeat motifs in HpaT proteins from *X. translucens*.

(A) List of HpaT amino acid sequences from five *X. translucens* strains, extracted from their genome sequences (DAR61454, GenBank acc. no. AMXY00000000; DSM 18974, acc. no. CAPJ01000000; CFBP 2541, acc. no. JWHD00000000; ART-Xtg29, acc. no. ANGG00000000; Xtu4699, acc. no. CP008714). Red characters represent the conserved 20-amino acid sequence motif. (B) Consensus sequence logo of the conserved repeat motif, based on the highlighted motifs in Panel A.
